# Structural Immaturity of Human iPSC-Derived Cardiomyocytes: *In Silico* Investigation of Effects on Function and Disease Modeling

**DOI:** 10.3389/fphys.2018.00080

**Published:** 2018-02-07

**Authors:** Jussi T. Koivumäki, Nikolay Naumenko, Tomi Tuomainen, Jouni Takalo, Minna Oksanen, Katja A. Puttonen, Šárka Lehtonen, Johanna Kuusisto, Markku Laakso, Jari Koistinaho, Pasi Tavi

**Affiliations:** ^1^A.I. Virtanen Institute for Molecular Sciences, University of Eastern Finland, Kuopio, Finland; ^2^Department of Biomedical Science, University of Sheffield, Sheffield, United Kingdom; ^3^Biophysics, Department of Physics, University of Oulu, Oulu, Finland; ^4^Institute of Clinical Medicine, Internal Medicine, University of Eastern Finland, Kuopio University Hospital, Kuopio, Finland

**Keywords:** human induced pluripotent stem cell-derived cardiomyocytes, excitation-contraction coupling, arrhythmias, repolarization, computational modeling

## Abstract

**Background:** Human induced pluripotent stem cell-derived cardiomyocytes (hiPSC-CMs) have emerged as a promising experimental tool for translational heart research and drug development. However, their usability as a human adult cardiomyocyte model is limited by their functional immaturity. Our aim is to analyse quantitatively those characteristics and how they differ from adult CMs.

**Methods and Results:** We have developed a novel *in silico* model with all essential functional electrophysiology and calcium handling features of hiPSC-CMs. Importantly, the virtual cell recapitulates the immature intracellular ion dynamics that are characteristic for hiPSC-CMs, as quantified based our *in vitro* imaging data. The strong “calcium clock” is a source for a dual function of excitation-contraction coupling in hiPSC-CMs: action potential and calcium transient morphology vary substantially depending on the activation sequence of underlying ionic currents and fluxes that is altered in spontaneous vs. paced mode. Furthermore, parallel simulations with hiPSC-CM and adult cardiomyocyte models demonstrate the central differences. Results indicate that hiPSC-CMs translate poorly the disease specific phenotypes of Brugada syndrome, long QT Syndrome and catecholaminergic polymorphic ventricular tachycardia, showing less robustness and greater tendency for arrhythmic events than adult CMs. Based on a comparative sensitivity analysis, hiPSC-CMs share some features with adult CMs, but are still functionally closer to prenatal CMs than adult CMs. A database analysis of 3000 hiPSC-CM model variants suggests that hiPSC-CMs recapitulate poorly fundamental physiological properties of adult CMs. Single modifications do not appear to solve this problem, which is mostly contributed by the immaturity of intracellular calcium handling.

**Conclusion:** Our data indicates that translation of findings from hiPSC-CMs to human disease should be made with great caution. Furthermore, we established a mathematical platform that can be used to improve the translation from hiPSC-CMs to human, and to quantitatively evaluate hiPSC-CMs development toward more general and valuable model for human cardiac diseases.

## Introduction

Human induced pluripotent stem cell-derived cardiomyocytes (hiPSC-CMs) have emerged as promising tools for cardiac research. In theory, hiPSC-CMs provide an accessible source of human cardiomyocytes without ethical and practical concerns that entail the use of human cardiac tissue or cells. From the experimental point of view hiPSC-CMs also solve the problems related with inter-species comparisons, thus enhancing the translation between basic research and clinical science. Moreover, since hiPSC-CMs retain the genetic identity of the individual donor, they enable generation of patient- and disease-specific cells that can be employed in procedures of personalized medicine. While hiPSC-CMs have become useful and popular cellular models to study mechanisms of human cardiac diseases (Blazeski et al., 2012; Iglesias-García et al., 2013; Eschenhagen et al., 2015) and for drug screening (Zeevi-Levin et al., 2012; Engle and Puppala, 2013), increasing attention has been paid to the question how similar they are compared with the adult human cardiomyocytes (Knollmann, 2013; Hwang et al., 2015; Kane and Terracciano, 2015).

Initially, justification for using hiPSC-CMs as a model for human cardiomyocytes came from the notion that they express most of the basic components underlying excitation-contraction coupling, membrane voltage regulation and even signaling cascades of cardiac myocytes (Ivashchenko et al., 2013; Karakikes et al., 2015). Furthermore, hiPSC-CMs have ion currents for depolarization (I_Na_, I_CaL_, I_f_) and repolarization (I_to_, I_Kr_, I_Ks_, I_K1_) of the membrane, which together produce, in subpopulations of hiPSC-CMs, action potential (AP) waveforms resembling that of human cardiomyocytes (Karakikes et al., 2015). hiPSC-CMs also express the central components of cardiac excitation-contraction (E-C) coupling, including L-type calcium channels and sodium-calcium exchangers (NCXs) (Ma et al., 2011; Yazawa et al., 2011; Zhang X.-H. et al., 2013; Uzun et al., 2016), as well as structures and proteins for sarcoplasmic reticulum (SR) calcium release and uptake (Germanguz et al., 2011; Itzhaki et al., 2011; Lee et al., 2011; Zhang X.-H. et al., 2013; Kim et al., 2015). However, the environment where all these components operate and interact differs substantially from the native or mature one. That is, compared to adult cardiomyocytes, hiPSC-CMs are much smaller and instead of having a rectangular shape they can also be round or polygonal (Hwang et al., 2015). Furthermore, iPSC-CMs lack a regular ultrastructure (Gherghiceanu et al., 2011; Itzhaki et al., 2011) and T-tubule network (Li et al., 2013; Kane et al., 2015). This results in poor co-localization of calcium channels and ryanodine receptors (RyRs) as well as non-uniform distribution of calcium release (Gherghiceanu et al., 2011; Rao et al., 2013). Therefore, in hiPSC-CMs the upstroke and decline rates of the whole-cell Ca^2+^ signals are substantially slower than in adult cardiomyocytes (Lee et al., 2011; Hwang et al., 2015). The emerging function has characteristics not shared with adult cardiomyocytes such as spontaneous beating, depolarized diastolic membrane potential, flat action potential duration restitution, slow Ca^2+^ signals and negative force-frequency relationship (Kane et al., 2015; Karakikes et al., 2015).

To evaluate quantitatively the translational potential of hiPSC-CMs, we constructed a mathematical model recapitulating their common *in vitro* features. Previous mathematical hiPSC-CM models focused mainly on the action potential morphology and sarcolemmal ion currents (Zhang H. et al., 2012; Paci et al., 2015). However, for a side-by-side comparison with detailed models of adult cardiomyocytes a more comprehensive hiPSC-CM model is required. One central feature to be included into such a model is a realistic representation of calcium dynamics, as well as cell-type-specific interplay between Ca^2+^ signals and membrane voltage. Employing the novel *in silico* hiPSC-CM model in standard simulations, sensitivity analysis and construction of a screenable database enabled us to (1) study the physiological properties of hiPSC-CM, (2) probe the biological relevance of the phenotypic variability of hiPSC-CMs reported *in vitro*, (3) compare properties side-by-side to human adult ventricular (Grandi et al., 2010) and atrial (Grandi et al., 2011) myocytes as well as to embryonic cardiomyocytes (Korhonen et al., 2010), and (4) explore to what extent different heart diseases can be recapitulated in hiPSC-CMs.

## Results

### Structural and functional characteristics of hiPSC cardiomyocytes

**Figure 1:**
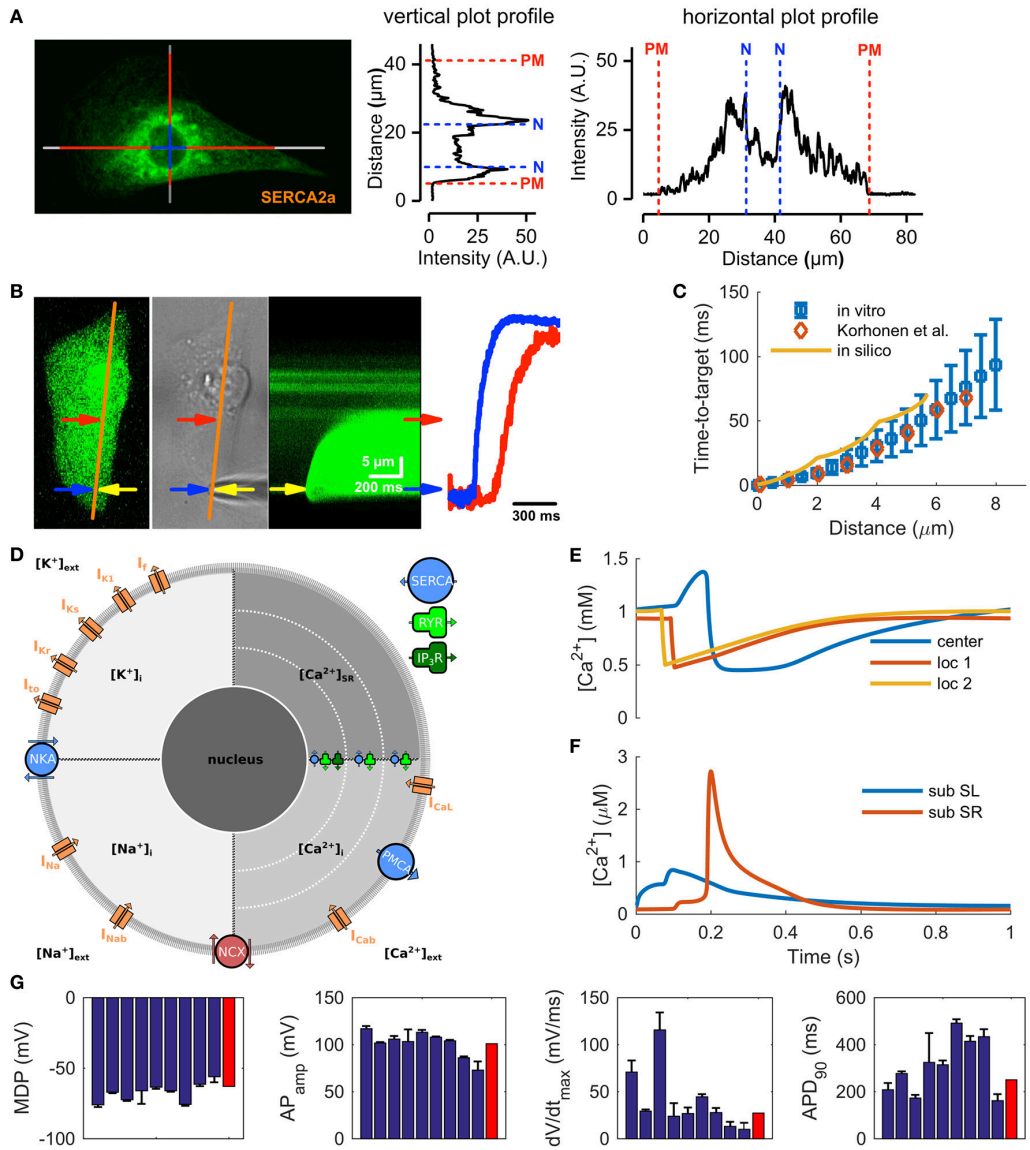
Calcium handling characteristics of hiPSC-CMs. **(A)** Representative confocal image from a hiPSC-CM immunostained with SERCA2a antibody and line plots illustrating the localization of the stain in the cells. **(B)** Recording of calcium diffusion in hiPSC-CM; from left to right: fluorescence reference image (orange line: line-scan place), photograph of the experimental setup (yellow arrow—patch pipette), line-scan recording obtained during injection of 1 μM Ca^2+^ solution from patch pipette, and line-scan profile at two different position (blue arrow—near the injection place, red—near central SR). **(C)** The time-to-target plots for average *in vitro* (mean ± SEM, *n* = 10) and *in silico* data are very similar to previously published mouse embryonic ventricular myocyte data from Korhonen et al. (2010). **(D)** Schematic presentation of the *in silico* hiPSC-CM model components and geometry, for the acronyms and detailed description of the model components, please see Methods section. Ca^2+^ concentrations in the central sarcoplasmic reticulum and two local release sites at 2 and 4 μm distance from the sarcolemma **(E)** and in the cytosol **(F)**, at 1 Hz pacing. **(G)** Comparison of AP characteristics in the hiPSC-CM model (red bars) to *in vitro* data (blue bars; mean ± SEM) listed in Supplementary information, Supplementary Table 4.

The structural immaturity affects calcium-induced calcium release (CICR) and limits the maximum cycle frequency by posing a substantial delay of about 50–90 ms between the central and peripheral calcium signals (Lee et al., 2011; Zhang G. Q. et al., 2013). While RyR and SERCA (SR Ca^2+^ ATPase) proteins are distributed throughout the cytosol (Ivashchenko et al., 2013) the bulk of the SR is located in the perinuclear region (Figure 1A and Supplementary Figure 1), with some extensions of SR throughout the cytosol (Itzhaki et al., 2011; Zhang X.-H. et al., 2013). In embryonic cardiomyocytes, with similar structures, the whole cell calcium transients are triggered from the perinuclear SR (Rapila et al., 2008) and the calcium propagation in the cytosol is boosted with local Ca^2+^ releases from SR extensions (Korhonen et al., 2010). According to our 2-D calcium diffusion measurements (Figure 1B) the speed of Ca^2+^ propagation in hiPSC-CMs (Figure 1C) is very similar to that of embryonic mouse myocytes both *in vitro* (Korhonen et al., 2010) and also when modeled *in silico* (Korhonen et al., 2010) (Figure 1D). Instead of pure diffusion, CICR underlies the “fire–diffusion–fire” propagation of the Ca^2+^ wave inside hiPSC-CMs.

Although hiPSC-CMs express a functional pacemaker current (I_f_), the density of the current is not sufficient on its own for spontaneous action potential (AP) generation (Kim et al., 2015). Spontaneous activation of hiPSC-CMs thus relies on interaction between the “Ca^2+^ clock” and the “membrane clock,” similar sinoatrial node cells (SANCs) (Maltsev and Lakatta, 2013). Indeed, stabilization (Kim et al., 2015) or inhibition (Kim et al., 2015; Zhang et al., 2015) of RyRs, as well as SERCA inhibition (Zhang et al., 2015) all reduce or abolish spontaneous activity in hiPSC-CMs. This suggests that automaticity depends on spontaneous Ca^2+^ release from SR initiated by activity of both RYRs and inositol-1,4,5-trisphosphate receptors (IP_3_Rs) (Itzhaki et al., 2011). That is, released calcium increases the cytosolic calcium concentration ([Ca^2+^]_i_) and triggers a depolarizing current via sodium-calcium exchanger (NCX) (Kim et al., 2015), serving as a trigger for AP. In line with previous reports (Fine et al., 2013; Zhang X.-H. et al., 2013), our data shows a strong expression (Supplementary Figure 1) and function (Supplementary Figure 2) of NCX in hiPSC-CMs. Furthermore, immunostaining of IP_3_R shows their strong presence around the nucleus (Supplementary Figure 1), confirming previous findings (Itzhaki et al., 2011).

**Figure 2:**
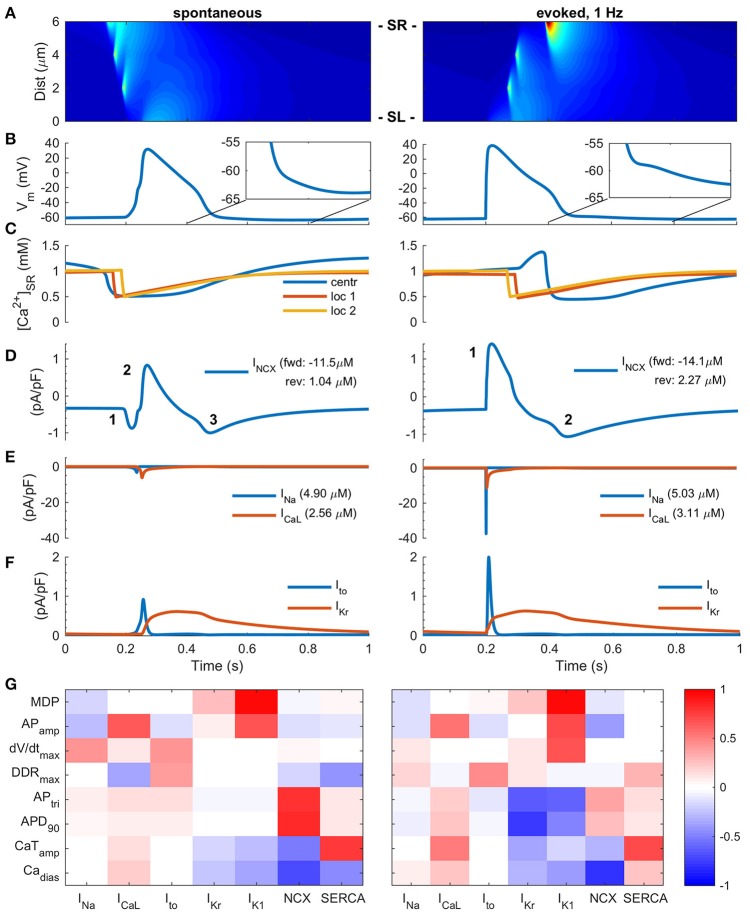
Two modes of Excitation-Contraction coupling in hiPSC-CMs. **(A)** Transient increase of intracellular Ca^2+^ concentration and Ca^2+^ diffusion in spontaneous (left) and paced (right) mode *in silico* measurements. The spatiotemporal representation is analogous to a line scan measurement *in vitro*. **(B)** AP in spontaneous (left) and paced, 1 Hz, (right) modes. **(C)** Ca^2+^ concentration in central sarcoplasmic reticulum and two local release sites at 2 and 4 μm distance from the sarcolemma. Sodium-calcium exchanger current **(D)**, sodium and calcium current **(E)**, and transient outward and delayed rectified potassium currents **(F)** in spontaneous (left) and paced (right) mode. The values in legends **(D,E)** indicate the ion flux integral over one AP cycle. Note: the direction of I_NCX_ in paced mode changes biphasically, while the spontaneous mode involves three phases. **(G)** Heatmap presentation of correlation coefficients of varied cellular components with eight different biomarkers in spontaneous (left) and paced (right) mode. MDP, minimum diastolic membrane potential; AP_amp_, amplitude of the action potential; DDR_trimax_, maximum diastolic depolarization rate; AP_tri_, action potential triangulation; APD_90_, action potential duration at 90% repolarization; Ca_dias_, minimum calcium concentration during diastole; CaT_amp_, amplitude of the calcium transient.

Based on this data we constructed the new model by first merging the cell geometry and ultrastructure of mouse embryonic myocyte model (Korhonen et al., 2010) with the membrane electrophysiology of a recent hiPSC-CM model (Paci et al., 2015) (Figure 1D). After this step, extensive model parameter fitting was done based on our own *in vitro* measurements and literature data (Supplementary Tables 1–3). The resulting model recapitulates the central immature characteristics of hiPSC-CMs, such as spontaneous activity (Kim et al., 2015) and inhomogeneous subcellular calcium distribution (Lee et al., 2011; Zhang G. Q. et al., 2013) (Figures 1E,F, 2A and Supplementary Figure 3). Moreover, basic characteristics of calcium signaling parameters, such as calcium transient and caffeine pulse decays and ratio between SR and SL calcium fluxes, are in line with the *in vitro* values (Supplementary Figures 2A–C). Finally, as the comparison of AP characteristics with literature data shows, the hiPSC-CM model is well within the range of reported *in vitro* values (Figure 1G, Supplementary Table 4).

### Mode of activation alters membrane currents and calcium cycling

A common feature of hiPSC-CMs separating them from mature atrial or ventricular CMs is their spontaneous beating. In literature, it appears that experimental results obtained in both modes of excitation, spontaneous and stimulated, are considered equivalent. Also in our *in silico* model, the AP morphology varies rather little depending on mode of activation (Figure 2B). AP amplitude and upstroke velocity are smaller in spontaneous vs. paced mode, while AP duration is almost identical. However, the fundamental ion currents and order of their activation are quite different depending on mode of activation (Figures 2D–F). In the spontaneous mode, the excitation trigger is the calcium release from the SR (Figures 2A,C), and thus the first membrane current to activate is I_NCX_ (Figure 2D). In the paced mode, the activation sequence is reversed and therefore the timing and dynamics of intracellular calcium is different, resulting in smaller calcium removal (18%, forward) and entry (54%, reverse) via NCX in spontaneous than paced mode. Depolarization of the membrane potential leads to activation of I_Na_, which then further leads to activation of I_CaL_. As the rate of depolarization is much slower in spontaneous vs. paced mode, the amplitude of I_Na_ is drastically smaller, −91%, (Figure 2E); a result of a phenomenon known as accommodation. The same phenomenon, affects I_CaL_ and I_to_ amplitudes as well, which are 45 and 54% smaller in spontaneous vs. paced mode, respectively (Figures 2E,F). The total sodium and calcium entries are only 3 and 17% smaller, respectively, in the spontaneous mode and the amplitude of the calcium transient (CaT) is only 10% smaller in the spontaneous vs. evoked mode.

Longer time course and altered timing of CaT in respect to AP also impacts the AP repolarization in spontaneously activated cells, enhancing calcium extrusion by NCX, which causes a depolarizing inward current at the late repolarization phase, thus creating a “tail” for the AP (Figure 2B). While this difference is subtle, it has a significant effect on excitability, as the availability of I_Na_, and thus refractoriness, has a very steep dependence on membrane potential in this voltage range (Skibsbye et al., 2016). NCX function is also strongly affected by the diastolic membrane potential, which is typically depolarized by up to 30–40 mVs in hiPSC compared adult CMs (Supplementary Figure 8). The detailed analysis show that forward mode is hampered and reverse mode enhanced at more depolarized potentials (Supplementary Figure 8F).

Sensitivity analysis of the hiPSC-CM model activated with either of the two modes demonstrates that if the cell is activated spontaneously, the AP parameters (triangulation, AP_tri_ and duration, APD_90_) depend more on NCX current and less on potassium currents (I_Kr_, I_K1_) compared to stimulated cells (Figure 2G). These findings highlight that the impact of any intervention aimed at modulating a specific component in hiPSC-CMs E-C coupling will depend on whether the cells are spontaneously active or electrically stimulated.

### Functional dissimilarities of hiPSC-CM compared to adult human cardiomyocytes

**Figure 3:**
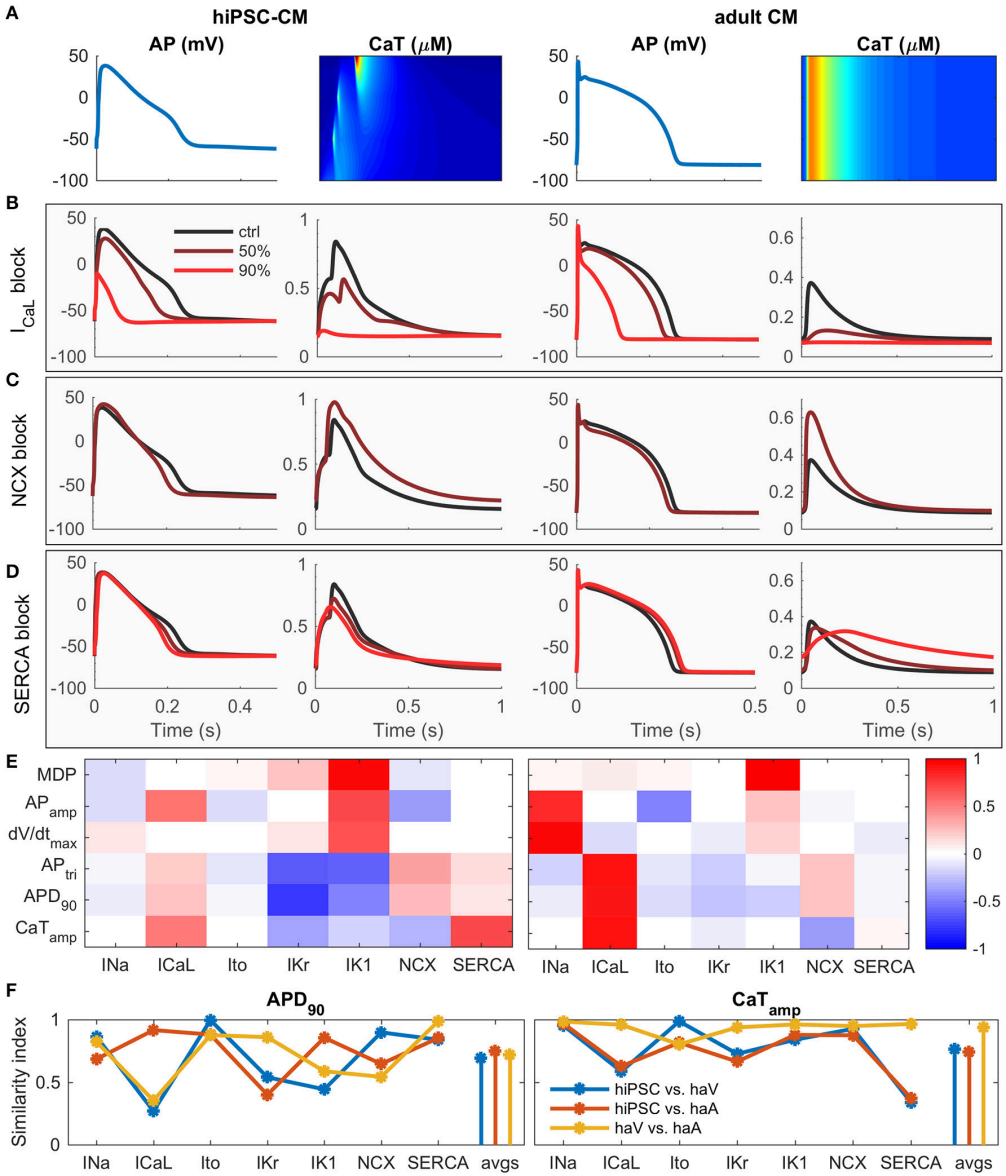
hiPSC-CM vs. adult CM phenotype *in silico*. **(A)** Comparison of AP and CaT in hiPSC (left) and adult (right) cardiomyocytes. Effect of 50 and 90% block of L-type calcium channel **(B)**, NCX **(C)** and SERCA **(D)** on AP and CaT in hiPSC (left) and adult (right) cardiomyocytes. For the NCX case, the results of 90% block are not shown, due to Ca^2+^ overload. **(E)** Correlation coefficients of sensitivity analysis. Same biomarkers as in Figure 2G. **(F)** Similarity index (sum of absolute difference of correlation coefficients) for APD_90_ and CaT_amp_ vs. seven key parameters, and average values (avgs). Comparisons made between hiPSC vs. human adult ventricular (haV) myocyte, hiPSC vs. human adult atrial (haA) myocyte, and human adult ventricular vs. atrial myocyte.

To elucidate the contribution of basic components to calcium cycling, we simulated the effect of 50 and 90% block of I_CaL_, NCX and SERCA (Figures 3A–D). While some of the changes are similar, the effect of I_CaL_ block on AP amplitude and duration is more dramatic in hiPSC-CMs (Figure 3B) and blocking of SERCA reduces the CaT amplitude much more in adult CM (Figure 3D). Sensitivity analysis (Figure 3E and Supplementary Figures 4A–D) indicates that the contribution of I_CaL_ on CaT is more significant in adult CMs. In hiPSC-CMs, APD is much more sensitive to changes in the rapid delayed rectified (I_Kr_) and inward-rectified (I_K1_) potassium currents, indicating that adult CMs have a stronger repolarization reserve. According to a sensitivity analysis based similarity index (Figure 3F), the AP of hiPSC-CM shares underlying mechanisms with both adult ventricular and atrial CMs, while the CaT dependencies are more similar between adult ventricular and atrial CMs than between hiPSC-CMs and either adult cell type. Interestingly, even though mouse embryonic cardiomyocytes lack two potassium currents (I_to_, I_Kr_), hiPSC-CMs appear to be functionally very similar with mouse embryonic myocytes as well (Supplementary Figure 4).

### Limited translation of pathology from hiPSC-CMs to adult cardiomyocytes

To assess the translational potential of hiPSC-CMs and directly compare hiPSC-CMs and adult cardiomyocytes to each other, we next implemented the modifications involved in Brugada Syndrome (BrS), Long QT Syndrome (LQTS) and catecholaminergic polymorphic ventricular tachycardia (CPVT).

**Figure 4:**
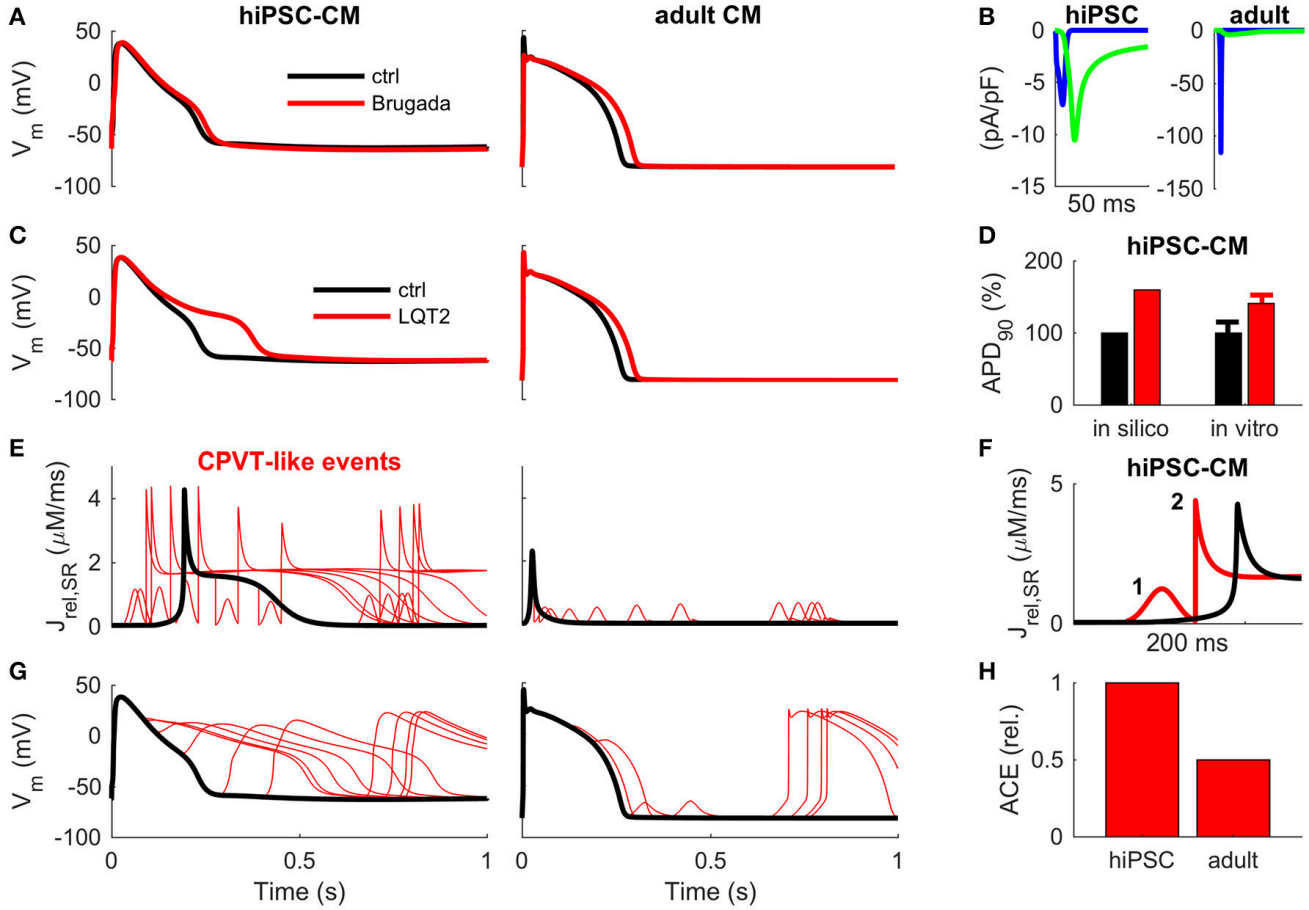
Translation of disease mechanisms from hiPSC-CM to adult CM *in silico*. **(A)** Effect of Brugada-associated Na_v_β1b/H162P mutation (Yuan et al., 2014) on AP morphology in hiPSC (left) and adult (right) cardiomyocytes. **(B)** Fast sodium (blue line) and L-type calcium (green line) currents underlying the depolarization phase of the AP in BrS in hiPSC (left) and adult (right) cardiomyocytes. Corresponding wildtype currents in hiPSC-CM are shown in Figure 2. **(C)** Effect of LQT2-associated c.A2987T KCNH2 mutation on AP repolarization in hiPSC (left) and adult (right) cardiomyocytes *in silico*. **(D)** AP duration *in silico* and *in vitro* mean ± SEM, as reported by Bellin et al. (2013). **(E)** Sarcoplasmic reticulum Ca^2+^ release (J_rel_) caused by random RyR openings (CPVT-like condition) in hiPSC (left) and adult (right) cardiomyocytes *in silico*. **(F)** Example of a primary (1) and secondary (2) J_rel_ in hiPSC-CM during one AP cycle. **(G)** Early and delayed after depolarizations in hiPSC (left) and adult (right) CM *in silico*. **(H)** Arrhythmogenic coupling efficiency (ACE) in hiPSC and adult CM, quantified as deviations in membrane voltage compared to control, is much stronger in hiPSC-CMs.

We simulated BrS by replicating a Na_v_β1b/H162P mutation (Yuan et al., 2014) (Figure 4A). In hiPSC-CM^BrS^ model variant, the normal activation of I_Na_ does not elicit an AP (Supplementary Figure 5A). However, it is possible to overcome the increased excitation threshold by using a stronger stimulus current, which depolarizes the membrane potential enough to activate the I_CaL_ (Supplementary Figure 5C). Interestingly, the AP morphology in the hiPSC-CM^BrS^ model differs very little from the control (Figure 4A). The peak of AP is reached 3.9 ms later and there is a slight deceleration of the late phase of AP repolarization (APD_90_ +10%, +25.2 ms). In adult CM, BrS blunts the initial spike of AP and slows the late repolarization slightly more (APD_90_ +14%, +35.8 ms). I_Na_ is so small in hiPSC-CM, and BrS reduces it even further to the extent, that I_CaL_ becomes the predominant depolarizing current (Figure 4B).

In a previously reported LQT2 mutation (c.A2987T KCNH2), the conductance of I_Kr_ was reduced by 33%, which resulted in increased action potential duration in hiPSC-CMs *in vitro* (APD_50_ +38% and APD_90_ +41%) (Bellin et al., 2013). The simulations with hiPSC-CM^LQT2^ model replicates those findings nicely (APD_50_ +29% and APD_90_ +60%, Figures 4C,D). However, running the same simulations with the adult CM model predicts substantially smaller changes (APD_50_ +13% and APD_90_ +12%, Figure 4C). This finding demonstrates that the repolarization reserve is much smaller in hiPSC-CMs compared to adult CMs, which also causes arrhythmias in the virtual hiPSC-CM^LQT2^ cell (Supplementary Figure 6).

Next, we simulated CPVT-type arrhythmias in hiPSC-CM and adult CMs with randomly timed SR Ca^2+^ releases via RyRs (Figures 4E–H). According to the simulations, due to the self-propagating nature of the hiPSC-CM calcium release (Figure 1), spontaneous RyR openings result in a complete release of SR calcium and whole cell CaT (Figures 4E,F). Moreover, as NCX has a larger role in calcium cycling of hiPSC-CMs (Figure 3), they are more prone to extra SR calcium release (J_rel_) induced membrane depolarizations CMs (Figure 4E) and have a higher arrhythmogenic coupling efficiency (ACE) than adult CMs (Figures 4G,H).

### Immature E-C coupling is the limiting factor of hiPSC-CM functional phenotype

**Figure 5:**
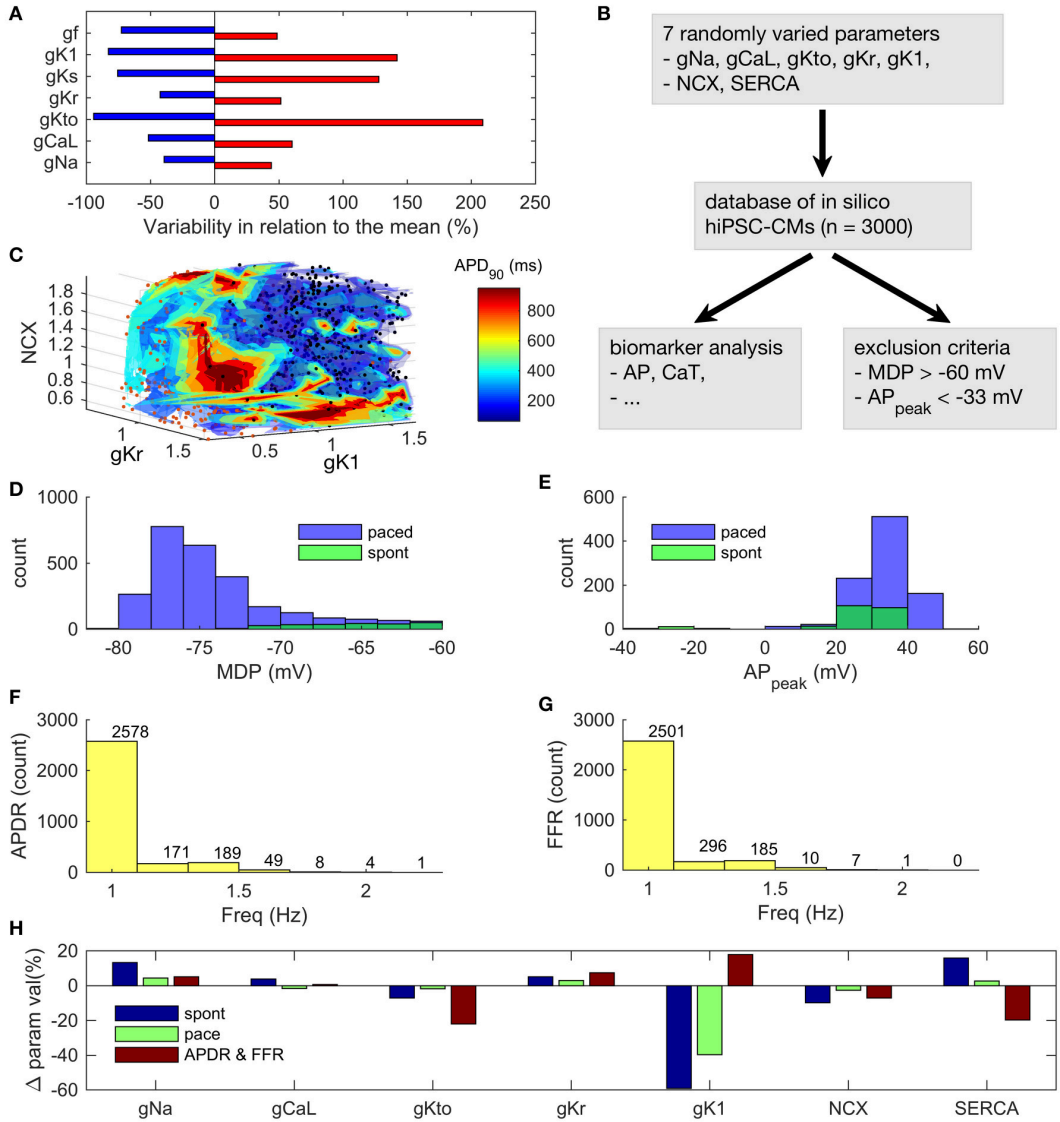
Analysis of an *in silico* database of hiPSC-CMs. **(A)** Ranges of variability in ion current conductances based on literature search. **(B)** Workflow in the database analysis. **(C)** 3D surface plot of APD_90_ as a function of three ion current parameters. The variable color of the data points is not quantitative or related to the color bar scale. Instead, it is just a way to increase the contrast of the dots against the surfaces. Histograms of maximum diastolic potential **(D)** and AP peak potential **(E)** in spontaneous and paced modes. Histograms of maximal frequency of AP duration restitution (APDR) **(F)** and force-frequency response (FFR) **(G)**. Maximum pacing frequency for monotonic APDR and FFR. **(H)** Relative parameter values in the (1) spontaneous and (2) paced modes, as well as, (3) in the monotonic APD restitution and force-frequency response subpopulations, compared to the whole database.

As hiPSCs are differentiated into hiPSC-CMs with variable techniques in different laboratories, they display a wide range of phenotypes (Figure 5A and Supplementary Tables 1–4). To analyse this huge variability, we created a database (Prinz et al., 2003) of 3,000 *in silico* hiPSC-CMs (Figure 5B), in which the parameter space was defined based on >25 publications (Supplementary Tables 1–4). As the time period of differentiation is variable in the published data, the resulting parameter space covers a wide field of theoretically possible hiPSC-CMs phenotypes. I_f_ and I_Ks_ conductances were not varied in the database, as in the *in vitro* ranges they had virtually no effect on the AP dynamics, please see section Database Simulations and Sensitivity Analysis for further details. We ran simulations both in the spontaneous and evoked/paced mode for all the virtual cells in the database. Some combinations of parameter values resulted in nonviable phenotypes (exclusion criteria described in section Materials and Methods). As a result, the number of viable *in silico* cells in database was reduced from 3,000 to 940 and 235 in the evoked (freq = 1 Hz) and spontaneous mode, respectively (Figures 5D,E).

**Figure 6:**
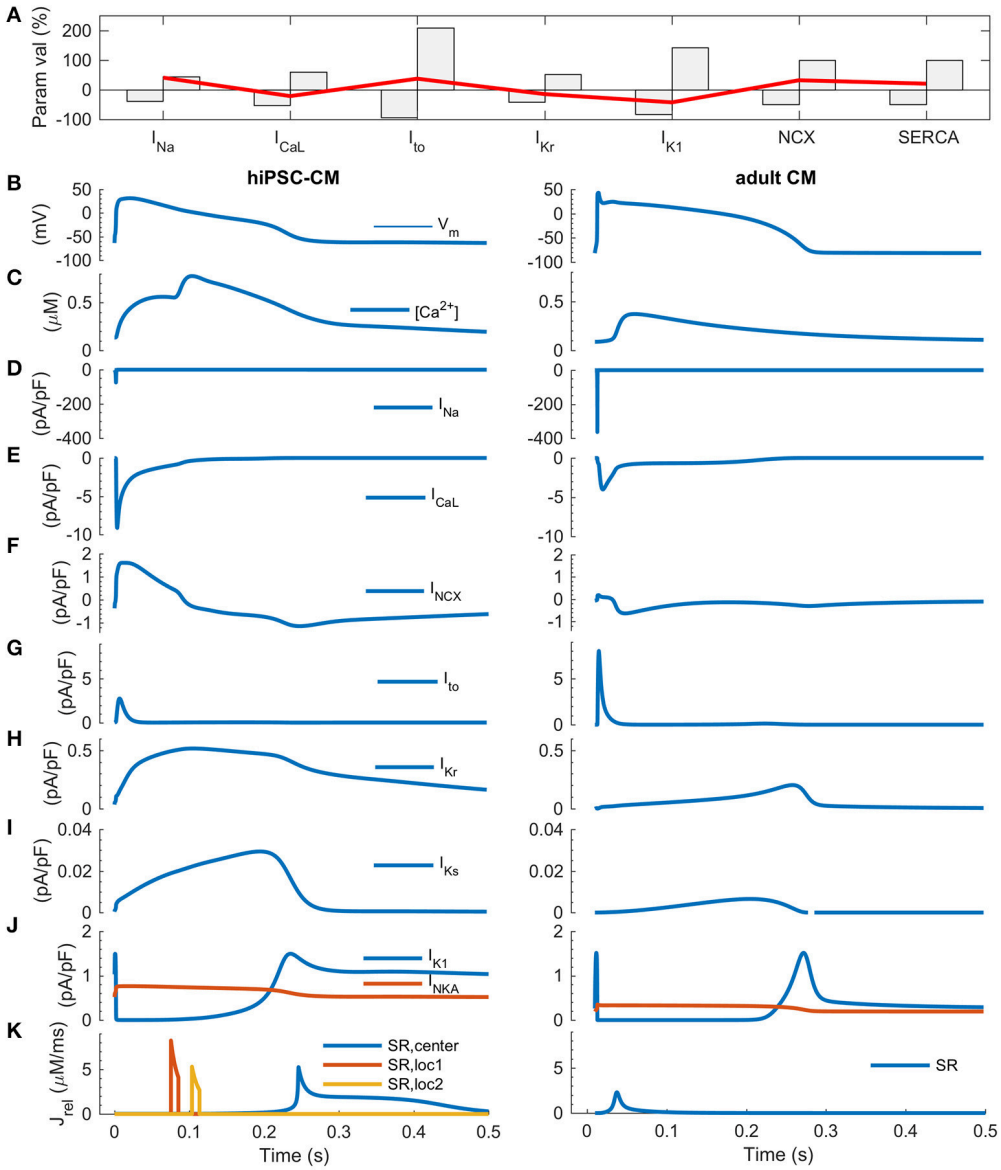
Comparison of ideal hiPSC-CM and adult CM *in silico*. **(A)** The most favorable parameter combination (red line) plotted with the full parameter range of the database on background (gray bars). Action potential **(B)**, calcium transient **(C)**, sodium current **(D)**, calcium current **(E)**, sodium-calcium exchanger current **(F)** transient outward, rapid and slow delayed rectified potassium currents **(G–I)**, inward rectified potassium current and sodium-potassium pump current **(J)**, and RyR-mediated Ca^2+^ release fluxes from the sarcoplasmic reticulum **(K)**. In the hiPSC-CM model, there are three spatially distinct release locations, as described in detail in Figure 1.

One of the key features of mature myocardium and cardiomyocytes is AP duration restitution (APDR): action potential becomes shorter, when the heart beat rate or the pacing frequency is increased (Figure 5 in Grandi et al., 2010). Thus, we explored the *in silico* database to see what kind of parameter value combinations would result in such a phenotype. In the database, it is possible to plot a biomarker such as APD_90_ (APD at 90% repolarization) as a function of the parameters that have been varied to build the population of models (Figures 5A,C). While there are a small number of cells that had a monotonically decreasing AP duration even up to ~2 Hz (Figure 5F), APD restitution is relevant only if there is a positive force-frequency relation (FFR) as well. Monotonically positive FFR was present up to 1.4 Hz (Figure 5G and Supplementary Figure 7) in the *in silico* cell database. Cross-comparison of the APDR and FFR subcollections showed that just 30 of 3,000 virtual cells recapitulated these basic features. The average parameter values of I_Na_ (+13%), I_Kr_ (+5%), I_K1_ (−59%), NCX (−10%) and SERCA (+16%) were statistically different (*p* < 0.05) in the spontaneously active subpopulation of 235 cells compared to the whole database (Figure 5H, blue bars). In the paced mode, the subpopulation of virtual cells with proper excitability (*n* = 940) had smaller, yet statistically significant deviations in the average parameter values for I_Na_ (+4%), I_Kr_ (+3%), I_K1_ (−40%), NCX (−3%) and SERCA (+3%). Surprisingly, cell variants recapitulating APDR+FFR (*n* = 30) had only a stronger I_K1_ (18%) and a weaker SERCA (−20%) (Figure 5H, red bars). From those 30 APDR+FFR *in silico* cells only two had an APD_90_ in the range of 250–300 ms. Interestingly, both of these ideal *in silico* hiPSC-CMs actually have about 40% smaller I_K1_ current density than on average in the database (Figure 6A), which contradicts the view that weak I_K1_ would be one of the limiting immature features of hiPSC-CMs (Meijer van Putten et al., 2015; Vaidyanathan et al., 2016). A side-by-side comparison shows that even though there is a rather good match in AP morphology with adult CM, the underlying ion currents and dynamics of the ideal hiPSC-CM still differ substantially from their mature counterparts (Figure 6). As in previous comparison scenarios, it appears that the ultrastructure-related differences in intracellular calcium handling cannot be overcome.

We also repeated the simulations with Brugada syndrome, LQT2 and CPVT-like model variants using a parameter combination that was found to be most favorable in the database analysis. The simulation shown in Supplementary Figure 9 indicate that the hiPSC-CM model with ideal parameters is slightly closer to the adult CM phenotype. That is, in the hiPSC-CM^BrS^ model, I_Na_ persists as the main depolarizing current. In the virtual hiPSC-CM^LQT2^ cell, increase of APD is also slightly smaller than in the model that has average parameters. However, the susceptibility to arrhythmogenic CPVT-like events is not changed.

## Discussion

Human iPSC-cardiomyocytes have emerged as popular cell models to study a variety of human cardiac diseases as well as for drug testing. In theory, hiPSC-CMs provide the first routinely accessible equivalent for native human cardiac myocytes, and solve the problems related to inter-species comparisons, which potentially hinder the development of therapies for human diseases. However, as more hiPSC-CM data is cumulating, concerns have risen regarding whether they are useful models for studying arrhythmias (Knollmann, 2013; Sinnecker et al., 2013) and electrophysiology (Han et al., 2014; Christ et al., 2015), or if their calcium signaling is comparable with that of adult cardiomyocytes (Hwang et al., 2015; Kane and Terracciano, 2015). To address these open questions, we have developed a novel mathematical model that recapitulates the functional characteristics of hiPSC-CMs, allowing us to compare them systematically and quantitatively with their adult counterparts.

### How does immaturity of hiPSC-CMs shape calcium dynamics?

According to our *in silico* analysis, many of the immature functional features are related to structures involved in intracellular calcium handling. Adult cardiomyocytes are relatively large cells, capable of generating strong, spatially homogenous Ca^2+^ signals at high frequency (Cannell et al., 1995; Bers, 2002). Although hiPSC-CMs express the same components for calcium handling, their Ca^2+^ signals are substantially slower and show much higher degree of spatial inhomogeneity (Li et al., 2013) (Figures 2, 3). This is not a surprise since spatiotemporal properties of calcium signals are not only affected by the efficiency of release and uptake but also Ca^2+^ propagation in the cytosol, which is relatively slow (diffusion constant ≈ 30 ms/μm) even at short (<15 μm) distances and is exponentially slower at longer distances (Korhonen et al., 2010). To overcome this biophysical obstacle, adult ventricular cardiomyocytes have unique cell membrane invaginations called T-tubules, which form a 3-D structure linking membrane and SR Ca^2+^ channels, thus minimizing the calcium diffusion distances in the cytosol (Cannell et al., 1995; Bers, 2002). Even though hiPSC-CMs have subcellular structures for enhancing Ca^2+^ propagation (Figure 1 and Supplementary Figure 1), the lack of T-tubules has profound functional effects. Firstly, there is a substantial delay of about 100 ms between the central and peripheral calcium signals (Figures 1, 2), which poses an absolute lower limit for the length of single E-C coupling cycle, and thus limits the maximal beating rate (Figure 5) (Korhonen et al., 2010). Secondly, this delay slows down the upstroke and decline rates of the whole cell CaTs in hiPSC-CMs, making them substantially slower than adult cardiomyocytes (Figure 3) (Lee et al., 2011; Hwang et al., 2015). This may appear as a minor detail, however, slower CaT kinetics change the timing of [Ca^2+^]_i_ -dependent currents during AP. Therefore, e.g., I_NCX_ contributes much more to the late AP repolarization in hiPSC-CMs than in adult CMs (Figure 3). In addition, compared to adult CMs, hiPSC-CMs rely more on sarcolemmal (I_CaL_, I_NCX_) than SR (RyR) calcium sources (Figure 3) (Lee et al., 2011). Importantly, larger I_NCX_ enhances the link between [Ca^2+^]_i_ and V_m_ and thus makes hiPSC-CMs more susceptible to after depolarization-triggered arrhythmias such as those triggering CPVTs (Figure 4). These features are important to consider, as hiPSC-CM should reflect the electrical stability/instability of adult human CMs, when they are used for drug testing or disease modeling.

### What are the functional implications of spontaneous vs. evoked mode in hiPSC-CMs?

While hiPSC-CMs are excitable and capable for CICR upon electrical excitation, one sign of their immaturity is that alongside with the normal E-C coupling they have the ability to generate spontaneous calcium oscillation for pacemaking (Figure 2 and Supplementary Figure 3) (Kane et al., 2015). Our detailed comparison of two modes of hiPSC-CMs activation (spontaneous vs. evoked) shows that there are substantial differences in the dynamics and magnitudes of ion currents, even though AP morphology was roughly similar in both modes (Figure 2). In the spontaneous mode, the rate of depolarization is much slower than in paced mode, during both triggering and upstroke phase of the AP. This causes a so-called accommodation phenomenon to happen in many of the ion channels: activation is so slow that inactivation starts to take place simultaneously. Therefore, the amplitudes of I_Na_, I_CaL_ and I_to_ are drastically smaller in spontaneous than paced mode. There is also a subtle difference in the final phase of AP repolarization: in the paced mode hiPSC-CMs display a very slow “tail” in the AP. As there is a very steep dependence of I_Na_ availability on membrane potential in this voltage range, this influences cardiac refractoriness, contrary to adult human ventricular CMs. It is important to consider these mode-dependent mechanisms, when utilizing hiPSC-CMs in experiments. For example, in drug screening, the effect of an ion channel blocker will be different in spontaneous vs. evoked mode of activation of the cells.

### How well do pathologies translate from hiPSC-CMs to adult cardiomyocytes?

Human-iPSC-CMs exhibit a heterogeneous phenotype, usually representing a mixed population of cells with diverse electrophysiological characteristics (Ivashchenko et al., 2013; Uzun et al., 2016). While the profile of ion channel expression is qualitatively similar to adult CM, the functional immaturity of hiPSC-CMs has raised concerns about their usability as disease models. Our analysis of BrS, LQT2 and CPVT scenarios confirms the doubts (Figure 4). For example, implementing a Brugada syndrome associated loss-of-function I_Na_ into the hiPSC-CM model reduces the excitability drastically and I_CaL_ becomes the main depolarizing current instead of I_Na_, which does not happen in adult CMs. The LQT2 simulation results demonstrate concretely the effect of a much smaller repolarization reserve in hiPSC-CMs, which together with the immature calcium handling makes them also much more sensitive to repolarization abnormalities, such as spontaneous SR Ca^2+^ release events in CPVT.

### What are the building blocks of a mature as possible hiPSC-CM phenotype?

Clearly, the electrophysiological differences between hiPSC-CMs and adult CMs complicate the comparison of these two cell types. Among the attempts aimed at reducing these differences, increased density of inward rectifying potassium current, I_K1_, has gained a lot of attention. As I_K1_ is important in stabilizing the resting membrane potential in adult cells, enhancing its magnitude has the potential to stop spontaneous beating of hiPSC-CMs (Meijer van Putten et al., 2015; Vaidyanathan et al., 2016). However, our database analysis suggests that modification of I_K1_, or any other ion current, is not enough to induce functional properties characterizing adult CMs such as action potential duration restitution or force-frequency relationship in hiPSC-CMs. Single modification of any of the varied parameters do not appear to solve these problems, which are mostly contributed by the immaturity of intracellular calcium handling.

Conclusive consensus of the physiological properties of hiPSC-CMs is lacking partly because the reported *in vitro* data is rather variable. As long as standardized experimental protocols do not exist, wealth of the variability originates form divergence of the maturity of cells used and the experimental conditions. Therefore, validation of hiPSC-CMs as a human cardiomyocyte model should take into account the variability as one of the features the hiPSC-CMs. Database analysis was used here to simulate the impact of the variability in the reported hiPSC-CM parameters to the phenotype of the cells. In practice, database analysis answers the question: what is the best possible hiPSC-CMs phenotype that the current methods can produce? Only 30 out of 3,000 parameter combinations produced a phenotype with fundamental physiological cardiomyocyte properties (APD restitution and FFR), and only in a very limited frequency range (up to ~1.5 Hz). Even though, the analysis was done in “ideal conditions”: the variables did not have any interdependence, i.e., all of them were varied independently, which is not likely the case in biological context. This finding also raises anticipation for the more advanced, and hopefully standardized, hiPSC-CM maturation protocols that are expected to deliver more mature-like cardiomyocytes.

### Limitations of the study

The chamber-specificity of hiPSC-CMs is a rather controversial topic, and there is no standard way for making this distinction. The most common way has been to use some sort of AP morphology index; however, this simplified technical approach has been rightfully criticized (Kane and Terracciano, 2017). Therefore, we opted not to implement separate atrial- and ventricular-like hiPSC-CM model versions. When reliable quantitative physiological criteria for determining the chamber-specificity have been established and taken into use, the developed hiPSC-CM model should be updated to have atrial- and ventricular-like versions accordingly.

We have not done a detailed comparison of the mechanisms of the “Ca^2+^ clock” and the “membrane clock” in hiPSC-CM vs. SANC. An in-depth analysis of the principal cellular components contributing to spontaneous activation would be very interesting and timely, as a model incorporating more *in vitro* human SANC data was recently published (Fabbri et al., 2017). However, this kind of a comparison is beyond the scope of this study.

Cellular signaling forms another layer of complexity to the regulation rhythmic activity in cardiomyocytes. As more *in vitro* hiPSC-CM data emerges on phosphatases, Ca^2+^/calmodulin-dependent protein kinase II, Phospholipase C pathway, guanylate cyclase, etc., the developed hiPSC-CM model needs to extended so that it can be employed in future research on those topics.

The spontaneous activation frequency of the novel hiPSC-CM model is 45.1 BPM, which is within the range of values reported *in vitro* (Supplementary Table 4). Accordingly, pacing experiments could not be simulated at 0.5 Hz frequency, which has been used in many *in vitro* studies. Instead, we used 1 Hz as the standard pacing frequency.

In the database simulations, the sample size of the spontaneously beating virtual cells was significantly smaller (*n* = 235) than the subpopulation that had proper excitability under pacing conditions (*n* = 940). However, in both scenarios, deviations of the same five parameter values (I_Na_, I_Kr_, I_K1_, NCX, and SERCA) from the average still reached statistical significance. Furthermore, the more focused analysis was done with the paced virtual cell population. So, the starting size of the database (*n* = 3,000) should not affect the conclusions made in that part of the study.

### Conclusion and future perspectives

The presented computational platform provides a quantitative tool for assessing hiPSC-CM properties, as well as comparing and translating hiPSC-CM findings to adult CMs. Our analysis suggests that the physiological properties of hiPSC-CMs differ from adult CMs in a way that warrants caution. As hiPSC-CMs show less robustness and greater tendency for arrhythmic events than adult CMs, translation of findings from e.g., particular ion channel mutation or pharmacological interventions is not straightforward. There is variability between different cell lines and culture conditions; however, the main bottleneck appears to be the structural immaturity of hiPSC-CMs. Recent efforts by multiple laboratories have succeeded in producing hiPSC-CMs with features, including e.g., functional T-tubule development (Parikh et al., 2017), more mature-like excitability (Lemoine et al., 2017) and contractile function (Mannhardt et al., 2016). This study provides a useful modeling framework for analyzing and improving those methods and techniques further.

## Materials and methods

### Derivation of induced pluripotent stem cells

Healthy fibroblast donor was recruited from Kuopio University Hospital (Kuopio, Finland; Approved by the committee on Research Ethics of Northern Savo Hospital district (license no 64/2014). Written informed consent was obtained from the donor. Skin biopsy derived fibroblasts were reprogrammed with CytoTune® iPS Sendai Reprogramming kit (Thermo Scientific, MA, USA) as previously described (Holmqvist et al., 2016), with slight modifications. Briefly, fibroblasts (1 × 10^5^) were transduced with 3 or 4 separate vectors including the four Yamanaka factors *OCT-3/4, KLF-4, SOX-2* and *c-MYC*. One week after transduction, 0.75 × 10^5^ cells were seeded on the top of mitotically inactivated (10 μg/ml mitomycin-C for 2.5 h in 37°C) human foreskin fibroblast feeder cells (CRL-2429, ATCC, Manassas, VA) growing in 10 cm petri dish. First colonies started to appear a week later, and they were re-seeded by picking up individual colonies. The pluripotency of created hiPSC line was assessed as in our earlier studies (Qu et al., 2013).

### Maintenance of iPS cells and cardiomyocyte differentiation

IPS cells were maintained in mTESR1 medium (Stem Cell Technologies, Canada) on human recombinant laminin-521 (Biolamina, Sweden) coated dishes at 37°C in a humidified 5% CO_2_ incubator. Cells were passaged with Tryple Express dissociation reagent (Thermo Fisher Scientific, MA, USA) 1–2 times a week just before cultures became confluent. Cells used in this study were between passages 5 and 23.

IPS cells were differentiated into cardiomyocytes using a protocol based on modulation of Wnt pathway (Lian et al., 2012). After dissociation into single cell suspension with Tryple Express, cells were plated on Matrigel (Corning Incorporated, NY, USA) coated dishes in mTESR1 medium. When the cells had reached full confluency, medium was changed to RPMI medium [RPMI 1640 Medium (Thermo Fisher Scientific, MA, USA) 1X B27 (Thermo Fisher Scientific, MA, USA), 100 U/mL penicillin-100 μg/mL streptomycin (Thermo Fisher Scientific, MA, USA)] supplemented with 12 μM CHIR99021 (Tocris, UK). After 24 h, CHIR99021 was removed and cells were kept in RPMI medium for 48 h. Next, cells were incubated in RPMI medium supplemented with 5 μM IWP2 (Tocris, UK) for 48 h, after which cells were kept in RPMI medium for 3–8 weeks, before preparing them for experiments.

For immunocytochemistry, patch-clamp and Ca^2+^ imaging spontaneously contracting hiPSC clusters were dissociated to single cells with a solution containing 2 mg/mL collagenase type II (Worthington, NJ, USA) and 2 mg/mL pancreatin (Sigma-Aldrich, MO, USA). Cells were plated in RPMI medium on glass coverslips coated with laminin (Sigma-Aldrich, MO, USA) at a density that allowed analysis of single cardiomyocytes. Cells were kept in RPMI medium for 3–7 days after plating, after which solution was changed to serum containing medium {Dulbecco's Modified Eagle Medium (Thermo Fisher Scientific, MA, USA) [10% fetal bovine serum (GE Healthcare Life Sciences, UT, USA), 100 U/mL penicillin-100 μg/mL streptomycin]}. Cells were kept in serum containing medium for another 3–10 days before immunological or live cell analysis.

### Electrophysiological recordings in isolated hiPSC cardiomyocytes

#### Patch-clamp experiments

All experiments were carried out at 37°C (TC2BIP, Cell MicroControls, USA). Coverslips with attached cells were transferred to the recording chamber (Cell MicroControls, USA, flow rate approx. 1–2 mL/min, chamber volume 0.4 mL) perfused with Dulbecco's modified Eagle medium plus glutamax I (DMEM, bubbled with 95% O_2_, 5% CO_2_). Whole-cell voltage-clamp (Axopatch 200B, Digidata 1440A, Molecular Devices Inc., USA) was used for Ca^2+^ current and current-clamp (I = 0) for action potential (AP) recordings. Patch electrodes (Harvard Apparatus, United Kingdom) were pulled and fire polished with Sutter P-97 (Sutter Instrument Company, Novato, CA). Patch electrodes for current recordings had resistances of 1.5–2.5 MΩ and 5–7 MΩ for AP recording and Ca^2+^ solution injection. Recordings were carried out after a membrane rupture of 5 min. The cell capacitance and series resistance were compensated electronically. The cells with an unstable or high access resistance were discarded. Under voltage clamp control cells were held at −80 mV. Membrane capacitance and resistance were estimated in response to a 5 mV pulse. The current amplitudes were normalized to cell capacitance. Recordings were carried out at a sampling rate of 10 kHz, and low-pass Bessel filtered at 5 kHz was used.

#### L-type Ca^2+^ current recordings

To characterize the L-type Ca^2+^ current (I_CaL_) we used the protocol described previously (Xu et al., 2011). The cells were perfused with Tyrode solution containing (in mM): 130 NaCl, 5.4 KCl, 1 CaCl_2_, 1 MgCl_2_, 0.3 Na_2_HPO_4_, 10 HEPES, and 5.5 glucose, pH 7.4 with NaOH, after establishment of whole-cell was switched to recording solution (solutions were bubbled with 100% O_2_). The internal solution contained (in mM): 110 CsOH, 90 aspartic acid, 20 CsCl, 10 tetraethyl ammonium chloride (TEA chloride), 10 HEPES, 10 EGTA, 5 Mg-ATP_2_, 5 Na_2_-creatine phosphate, 0.4 GTP-Tris, 0.1 leupeptin (pH 7.2 with CsOH) and bath solution: 125 N-methyl-glucamine, 5 4-aminopyridine, 20 TEA chloride, 2 CaCl_2_, 2 MgCl_2_, 10 glucose, 10 HEPES (pH 7.4 with HCl). After an initial 1-sec prepulse at −40 mV, Ca^2+^ currents were elicited using 200-ms voltage steps from −30 to +50 mV in 10-mV increments. Voltage-dependence of inactivation was assessed by holding cells at various potentials from −40 to +10 mV for 2 s, followed by a 100-ms test pulse to +10 mV.

#### AP recordings

Action potentials were elicited by a 1-ms current injection, and recorded using the current-clamp mode (Yang et al., 2005). Only well attached hiPSC-CMs with visible spontaneous contractions we included in the analysis. The cells that had APs without overshoots (peak amplitude at positive membrane potential) or/and with prominent membrane voltage drop were discarded. The intracellular solution contained (in mM): 120 K-aspartate, 8 KCl, 1 MgCl_2_, 7 NaCl, 2 Na_2_-phosphocreatine, 5 Mg-ATP, 0.3 Na-GTP, and 10 HEPES, (pH 7.2 with KOH) and the bath solution was DMEM.

### Confocal calcium imaging

Calcium imaging was performed as previously described (Mutikainen et al., 2016). Cardiomyocytes were loaded with Fluo-4-acetoxymethyl (AM)-ester (2 μM, Invitrogen) in DMEM for 20 min in an incubator (37°C, 5% CO_2_) and then coverslips with attached cells were placed into the recording chamber. Experiments were carried out after a period of 20 min to allow deesterification of the dye. [Ca^2+^]_i_ measurement was performed with a confocal inverted microscope (FluoView 1000; Olympus, Japan). To measure myocyte calcium [Ca^2+^]_i_ transients, the cells were excited at 488 nm and the emitted light (500–600 nm) was collected through water immersion 60X objective lens, using the line-scan mode. To stimulate the cells, myocytes were stimulated with 1-ms voltage square pulses (Grass stimulator, S48) 50% over the excitation threshold through platinum electrodes. In some experiments, caffeine (10 mM, Sigma) was applied directly to the studied area with a local perfusion manifold (Cell MicroControls, USA). Fluo-4 fluorescence intensity is expressed as an F/F_0_-ratio, where F is the background subtracted fluorescence intensity and F_0_ is the background subtracted minimum fluorescence value measured from each cell at rest. The images were analyzed with FluoView and ImageJ (imagej.nih.gov/ij/) softwares.

#### Calcium injections for measuring diffusion

The whole-cell voltage-clamp mode was used for 1 μM Ca^2+^ solution injection into fluo2-loaded cells (5 μM; TEFLabs, Inc; Austin, USA). The pipette was attached to a membrane with a Giga-seal (>3GΩ). Patch-pipettes were filled with injection solution containing (in mM): 0.84 CaCl_2_, 130 KCl, 5 Na2-creatine phosphate, 5 Mg-ATP_2_, 1 EGTA, 10 HEPES, pH 7.2 with KOH, 1.042 μM free Ca^2+^ (Smith et al., 1984). Injection of pipette solution was performed immediately after cell membrane rupturing, as previously described (Korhonen et al., 2010), by a 3 ms pressure pulse through pipette holder with microinjector (Picopritser II, Parker Instrumentation). The cells were held at a −70 mV.

### Immunofluorescence labeling

Cells cultured on glass coverslips were washed once with Dulbecco's phosphate buffered saline (PBS, Sigma-Aldrich, MO, USA), fixed with 4% paraformaldehyde (in PBS) for 5 min and permeabilized with 0.5% Triton-X (in PBS) (Sigma-Aldrich, MO, USA) for 10 min. Coverslips were washed twice with PBS for 5 min after which they were incubated with blocking buffer [PBS (10% FBS, 0.05% Triton-X)] for 1 h. After blocking, cells were incubated with primary antibody in blocking buffer for 1 h, washed, and incubated with secondary antibody in blocking buffer for 1 h. All labeling steps were performed at room temperature. Nuclei were stained with 14.3 μM DAPI (Thermo Fisher Scientific, MA, USA). Primary antibodies used were: Serca2 ATPase (mouse monoclonal, ab2861, Abcam, UK) (1:500 dilution), Ryanodine receptor (mouse monoclonal, ab2827, Abcam, UK) (1:100), IP_3_ receptor type 1 (rabbit polycolonal, ab111087, Abcam, UK) (1:100) and Sodium/calcium exchanger (mouse monoclonal, MA3-926, Thermo Fisher Scientific, MA, USA) (1:100). Secondary antibodies were, anti-Mouse IgG (goat polyclonal, A11001, Thermo Fisher Scientific, MA, USA) (1:750) and anti-Rabbit IgG (goat polyclonal, A21245, Thermo Fisher Scientific, MA, USA) (1:750).

### Statistics

Data and statistical analyses were made using Origin9 software (OriginLab Corp., Northampton, MA, USA).

### Novel *in silico* hiPSC-CM model

The usefulness of mathematical modeling as a tool requires that the fundamental properties of the cell are recapitulated accurately. In the special case of hiPSC-CMs, this means that the model needs to have a proper representation of the mechanisms of automaticity: the so-called calcium and membrane clocks. Previous mathematical hiPSC-CM models focused mainly on the action potential morphology and sarcolemmal ion currents (Zhang H. et al., 2012; Paci et al., 2015) and did not recapitulate the spontaneous SR Ca^2+^ release, which is a central feature of hiPSC-CMs. Accordingly, we developed a new *in silico* model that merges the cell geometry and immature intracellular calcium handling of a previously published mouse embryonic ventricular myocyte model (Korhonen et al., 2010) with the membrane electrophysiology of a recent hiPSC-CM model (Paci et al., 2015), using the ventricular-like variant of that model (Figure 1D).

As shown by the time-to-target analysis of intracellular Ca^2+^ diffusion (Figure 1C) and cell size comparison (Supplementary Figure 2I and Supplementary Table 3), the geometry and calcium handling of the embryonic cell model is applicable to hiPSC-CM modeling as well. Furthermore, to properly recapitulate the mechanisms of automaticity, three components of the electrophysiology part of the model were modified to be better in line with *in vitro* data (Supplementary Figure 2). Firstly, new formulation (Skibsbye et al., 2016) was adopted for the I_Na_ and fitted to the Ma et al. (2011) *in vitro* hiPSC-CM data. Secondly, the I_CaL_ formulation with a new one (Koivumäki et al., 2014), and fitted the properties to our own *in vitro* data. Thirdly, activation kinetics of the funny current (I_f_) were modified to be better in line with Sartiani et al. (2007) *in vitro* data.

The virtual hiPSC-CM model (Figure 1D) accounts for

- sarcolemmal fast and background sodium currents (I_Na_ and I_Nab_),
- sarcolemmal L-type and background calcium currents (I_CaL_ and I_Cab_),
- sarcolemmal potassium currents (I_to_, transient outward; I_Kr_, rapid delayed-rectified; I_Ks_, slow delayed-rectified; I_K1_, inward-rectified; I_f_, hyperpolarization activated),
- ion pumps and exchangers (SERCA, sarcoplasmic reticulum calcium ATP-ase; PMCA, plasmalemmal Ca^2+^ ATP-ase; NCX, sodium-calcium exchanger; NKA, sodium-potassium ATP-ase), and
- sarcoplasmic reticulum Ca^2+^ release channels (RyR, Ryanodine receptor; IP_3_R, Inositol trisphosphate receptor).

Importantly, the novel *in silico* model recapitulates the mechanisms of automaticity, as reported in previous *in vitro* studies (Supplementary Figure 3). That is, a full block of sodium calcium exchanger (NCX) stops the spontaneous activity, while a partial I_f_ block (corresponding to 3 μM Ivabradine) has virtually no effect on automaticity (Kim et al., 2015). Recapitulating the cell-type-specific interplay between Ca^2+^ signals and membrane voltage is a central requirement for making comprehensive *in silico* comparisons between adult CMs and hiPSC-CMs, both in physiological and pathophysiological scenarios.

The parameter values for the main ion currents were defined based on an exhaustive literature search, the results of which are shown in Figure 5 and in the supplementary material (Supplementary Tables 1–4). The parameter set was frozen on 06/2016. The chamber-specificity of hiPSC-CMs is rather controversial topic, as there is no standard way for making this distinction (Kane and Terracciano, 2017). Furthermore, many of the publications do not make a distinction, so we decided not to do it either. This way we were able to include much more *in vitro* data for model parameterization.

The basic outputs of the average model, in spontaneous and evoked mode, are shown in Figures 1, 2, 6B–K.

Source code of the developed hiPSC-CM model will be freely available via email upon request, as well as distributed via the ResearchGate networking portal in Matlab format.

### Experimental protocols *in silico*

Unless stated otherwise, all the *in silico* results were obtained either at spontaneous or stimulated steady-state. In the stimulated mode, action potentials were evoked by using a current pulse, whose amplitude was 1.5-times the threshold and length 0.5 ms. In the voltage clamp experiments (I_Na_ and I_CaL_), we used protocols and conditions identical to the *in vitro* measurements.

The following biomarkers were measured from the *in silico* data:

- MDP: minimum (negative) diastolic membrane potential
- AP_peak_: peak potential of the action potential
- AP_amp_: amplitude of the action potential
- APD_30_: action potential duration at 30% repolarization
- APD_90_: action potential duration at 90% repolarization
- AP_tri_: action potential triangulation = (APD_90_ − APD_30_)/APD_90_
- Ca_dias_: minimum Ca^2+^ concentration during diastole
- CaT_amp_: amplitude of the calcium transient

Caffeine application experiments were simulated by holding the RyR constantly open (50%), while blocking LTCC and SERCA. The time-to-target analysis of intracellular Ca^2+^ diffusion was done from data obtained while holding the virtual cell in voltage clamp (V_hold_ = −80 mV). Time for Ca^2+^ diffusion to a certain distance was defined with a threshold of 220 nM. A 2 μM Fluo-4 (Kd = 335 nM) was included in the cytosolic Ca^2+^ buffer composition. To mimic the Ca^2+^ puff from the patch pipette, the L-type Ca^2+^ channel held constantly open [ICaL = 0.5 ^*^ (Vm - ECa)] for 10 ms.

To define the dependence of NCX function on diastolic membrane potential (Supplementary Figure 8), a standard current stimulus pulse was used together with steadily changing baseline. During the 60-s protocol diastolic membrane potential was depolarized from about −80 to about −60 mV.

To elucidate the contribution of basic calcium cycling components, we simulated the effect of 50 and 90% block of I_CaL_, NCX and SERCA (Figures 3B–D), both in the novel hiPSC-CM model and in the previously published human ventricular (Grandi et al., 2010) CM model. The blocking effects were implemented by reducing maximum conductance/current/turnover rate by either 50 or 90% from the control parameter value.

### Database simulations and sensitivity analysis

We used both a conventional sensitivity analysis and the so-called database approach or population-based method for exploring biological robustness and variability. For the sensitivity analysis, we varied the parameter values for the maximum conductances of I_to_, I_Kr_, I_K1_, I_CaL_, and I_Na_, as well as maximum transport rates of SERCA and NCX by ±10% (*n* = 14). Correlation coefficients were calculated using Matlab's built-in function *corrcoef*. Similarity index for APD_90_ and CaT_amp_ was calculated as a sum of the relative contribution of the seven cellular components on the chosen set of biomarkers (APD_90_ and CaT_amp_).

In the database approach, we varied the same seven key parameters in the model according to available literature *in vitro* data (Figure 5, Supplementary Table 1). This experimentally-calibrated approach of creating a population of models was introduced by Prinz et al. (2003) in the context of *in silico* studies of neurons, and later applied also in computational cardiac studies by e.g., Romero et al. (2009).

We excluded the hyperpolarization activated or funny current (I_f_) and slow delayed rectified potassium current (I_Ks_) from the group of varied parameters. This was done to limit the computational load of database simulation, which is exponentially proportional to number of varied parameters. Also, the exclusion was physiologically justified, as changing I_f_ and I_Ks_ conductances in the *in vitro* ranges had virtually no effect on the AP dynamics. Instead, we studied I_f_ contribution separately to test if the current is large enough to contribute to spontaneous activity (Supplementary Figure 3E).

Database simulation were carried out with three protocols:

- In the spontaneous mode, simulations were run for 260 s and the last 10 s were saved for analysis.
- In the stimulated mode, simulations were run for 260 s at 1 Hz pacing and the last 5 APs were saved for analysis.
- In the APD restitution and FFR experiment, simulations were run for 60 s at each pacing frequency (1, 1.2, 1.4, 1.6, 1.8, 2.0, 2.2 Hz) and the last AP was saved for analysis.

All simulations were started from the control 1 Hz pacing steady-state. The 260-s simulation duration was justified by the estimate that the time constants for settling of [Na^+^]_i_ and [K^+^]_i_ was about 130 s in the model. In the database simulations, we used a slightly larger current pulse (amplitude 2-times the threshold) to evoke action potentials. APD restitution was measured as the shortening of APD_90_ and FFR as the increase of CaT amplitude (surrogate measure of force, as the model does not include the description of the contractile element).

### Pathological *in silico* model variants

We chose Brugada Syndrome (BrS), Long QT Syndrome (LQTS) and catecholaminergic polymorphic ventricular tachycardia (CPVT) as the three principal types of inherited arrhythmia that have electrical origin and manifest as abnormalities in excitation, repolarization and depolarization.

Multiple ion channel mutations are associated with BrS. We chose a Na_v_β1b/H162P (Yuan et al., 2014) mutation as an example case, in which the properties of I_Na_ are altered so that (1) current amplitude is reduced by 48%, (2) steady-state inactivation curve is shifted by 6.7 mVs toward negative potentials, and (3) slow and fast recovery from inactivation are 75 and 46% slower, respectively.

To quantify the effect of LQT2-associated c.A2987T KCNH2 mutation on AP repolarization in both hiPSC and adult cardiomyocytes, conductance of rapid delayed inward rectifying potassium current (I_Kr_) was decreased by 33%, based on the *in vitro* data from Bellin et al. (2013).

CPVT-like conditions were elicited both in hiPSC and adult cardiomyocytes, by forcing random RyR openings and subsequent calcium releases from the SR. Early and delayed afterdepolarizations caused by forced random RyR openings (Figure 4). Arrhythmogenic coupling efficiency (ACE) was quantified as deviations in membrane voltage compared to control.

### Human adult cardiomyocyte *in silico* models

To compare the hiPSC phenotype and human adult cardiomyocytes, we used the previously published ventricular (Grandi et al., 2010) and atrial (Grandi et al., 2011) cell models. In the BrS, LQT2 and CPVT-like model variants, the same pathology related modifications of model parameters were implemented as in the hiPSC-CM model. We chose to use ventricular and atrial CM models from the same Grandi et al. model familiy, so that a direct comparison between human adult ventricular and atrial myocytes was possible.

## Author contributions

Conception and design of the experiments: PT and JTK. Collection, analysis and interpretation of data: JTK, NN, TT, JT, JKu, ML, JKo, and PT. Drafting the article or reviewing it critically for important intellectual content: JTK, NN, TT, JT, JKu, ML, JKo, and PT. All authors approved the final version of the manuscript.

### Conflict of interest statement

The authors declare that the research was conducted in the absence of any commercial or financial relationships that could be construed as a potential conflict of interest.
