# Supplementary material for: Structural Immaturity of Human iPSC-Derived Cardiomyocytes: In Silico Investigation of Effects on Function and Disease Modeling
Source: Front Physiol. 2018 Feb 7;9:80. doi: 10.3389/fphys.2018.00080 (PMC5808345; doi:10.3389/fphys.2018.00080)
Supplement: Supplementary file 1 [file DataSheet1.pdf]

frontiers

Supplementary Material

Structural immaturity of human iPSC-derived cardiomyocytes: *in silico* investigation of effects on function and disease modelling

Jussi T. Koivumäki, Nikolay Naumenko, Tomi Tuomainen, Jouni Takalo, Minna Oksanen, Katja A. Puttonen, Šárka Lehtonen, Johanna Kuusisto, Markku Laakso, Jari Koistinaho, Pasi Tavi*

*** Correspondence:** pasi.tavi@uef.fi

1 Supplementary Figures

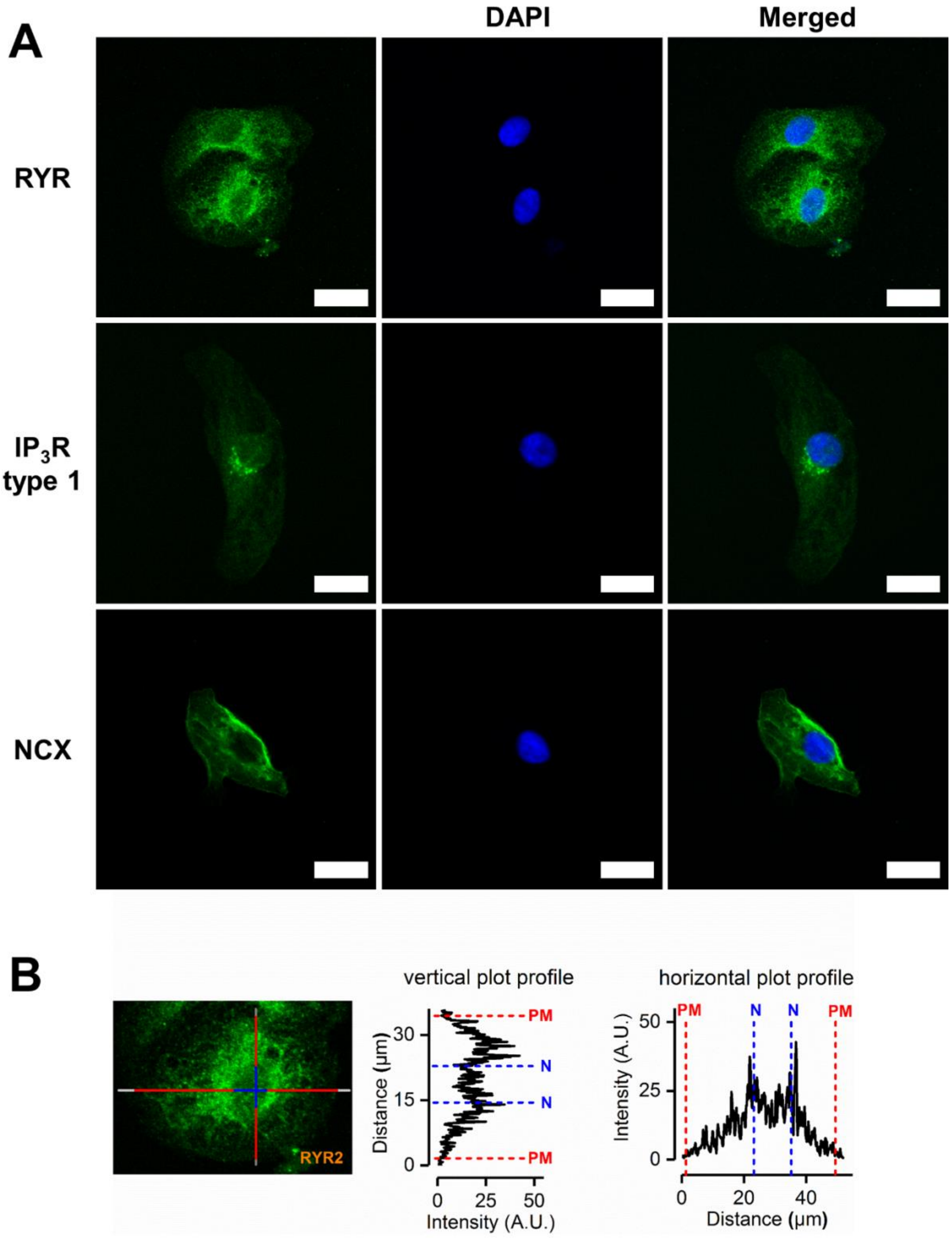

Supplementary Figure 1 | Immunolabelling of calcium handling proteins. (A) Representative images from hiPSC-CM labelled with antibodies detecting calcium handling proteins and nuclear

stain DAPI. Scale bar width 20 µm. RyR = Ryanodine receptor; IP_3R type 1 = Inositol trisphosphate receptor type 1; NCX = Sodium/calcium exchanger. **(B)** Similar to SERCA (SR Ca^{2+} ATPase), the RyR proteins are distributed throughout the cytosol, with a higher concentration in the perinuclear region (compare to Figure 1A). Stainings also display a positive IP_3R immunosignal that is strongly distributed around the nucleus.

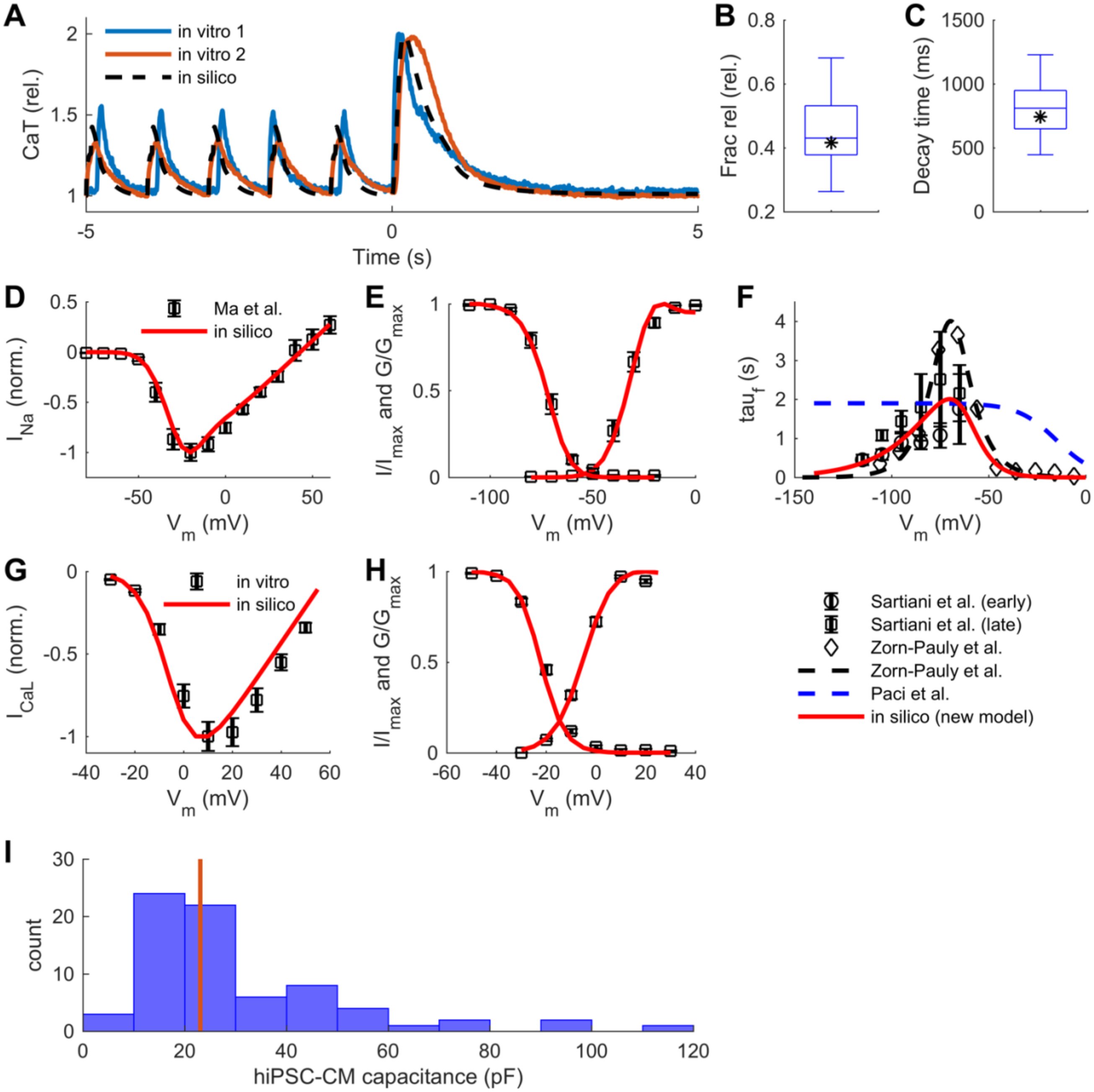

Supplementary Figure 2 | Fitting of reformulated ion currents and central model outputs. (**A**) Representative *in vitro* CaT traces compared to *in silico* data, during a caffeine experiment (caffeine application at time = 0). (**B-C**) Properties of sarcoplasmic reticulum and sarcolemmal calcium handling explored *in vitro* (boxplots; n=36): fractional SR calcium release and 66% decay time of calcium transient. The corresponding *in silico* values (black stars), 0.4158 and 743.2 ms, are well within the measured range of variability. Bars in the boxplots are at [min>1, 25, 50, 75, max<99] percentiles. (**D-E**) Fitting of new I_{Na} formulation to Ma et al. *in vitro* data (Ma et al., 2011). (**F**) Refitting the Paci et al. (Paci et al., 2012) I_f time constant to Sartiani et al. (Sartiani et al., 2007) *in vitro* data. (**G-H**) Fitting of new I_{CaL} formulation to own *in vitro* data (n=34). (**I**) Distribution of hiPSC-CMs size (capacitance) *in vitro* (n=88), size of the virtual cell is indicated with the red line.

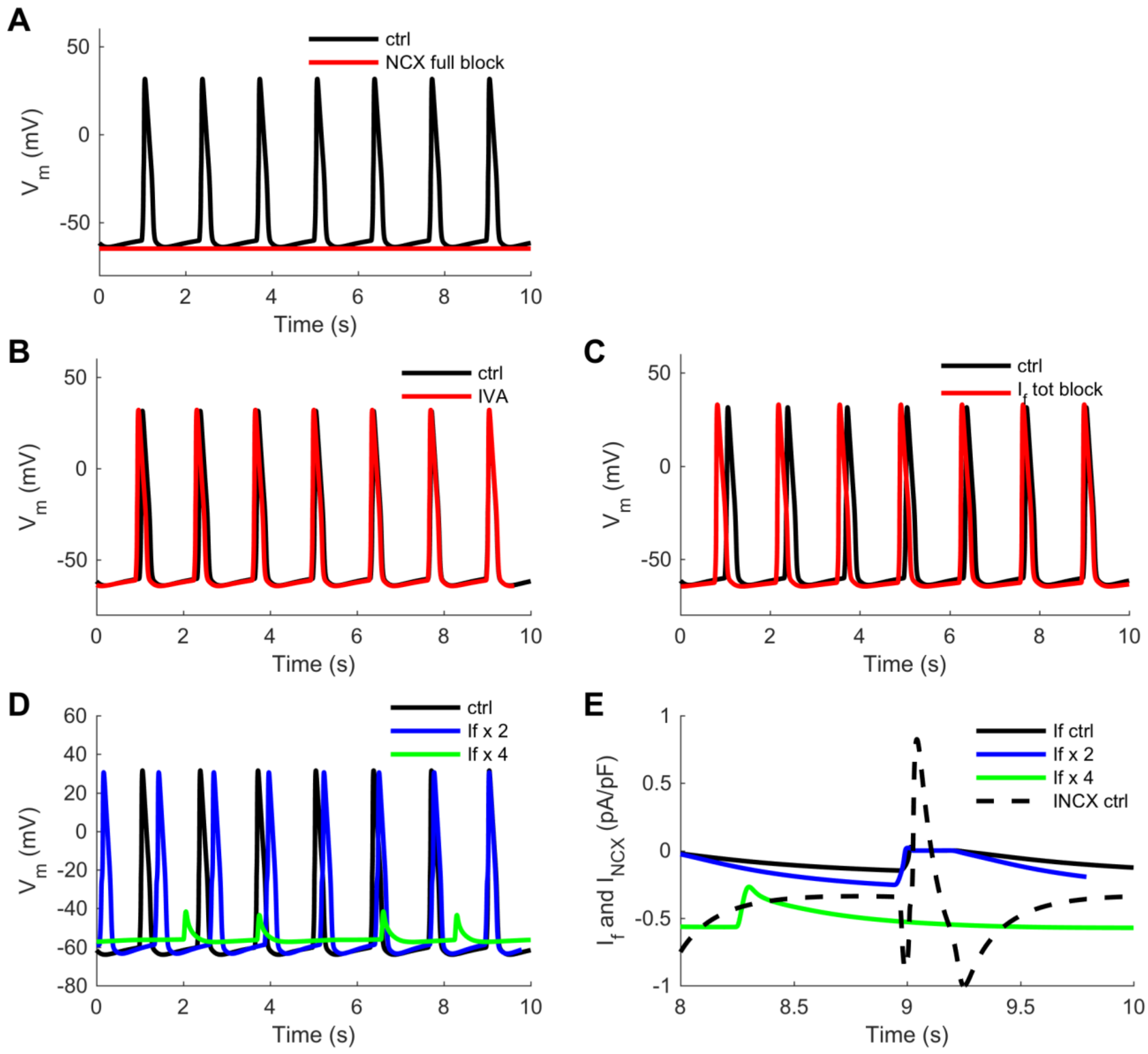

Supplementary Figure 3 | Mechanisms of automaticity investigated *in silico*. (**A**) Effect of 100% block of NCX. (**B**) Effect of Ivabradine (3 µM), simulated as 41% block I_f as in (Yaniv et al., 2012). (**C**) Effect of 100% block of I_f. (**D**) Effect of I_f overexpression (2- and 4-fold). (**E**) Traces of I_f in control and overexpression compared to NCX current in control. Even with 2-fold overexpression of I_f the current amplitude (green line) is only a fraction of the depolarising current mediated by NCX (dashed black line).

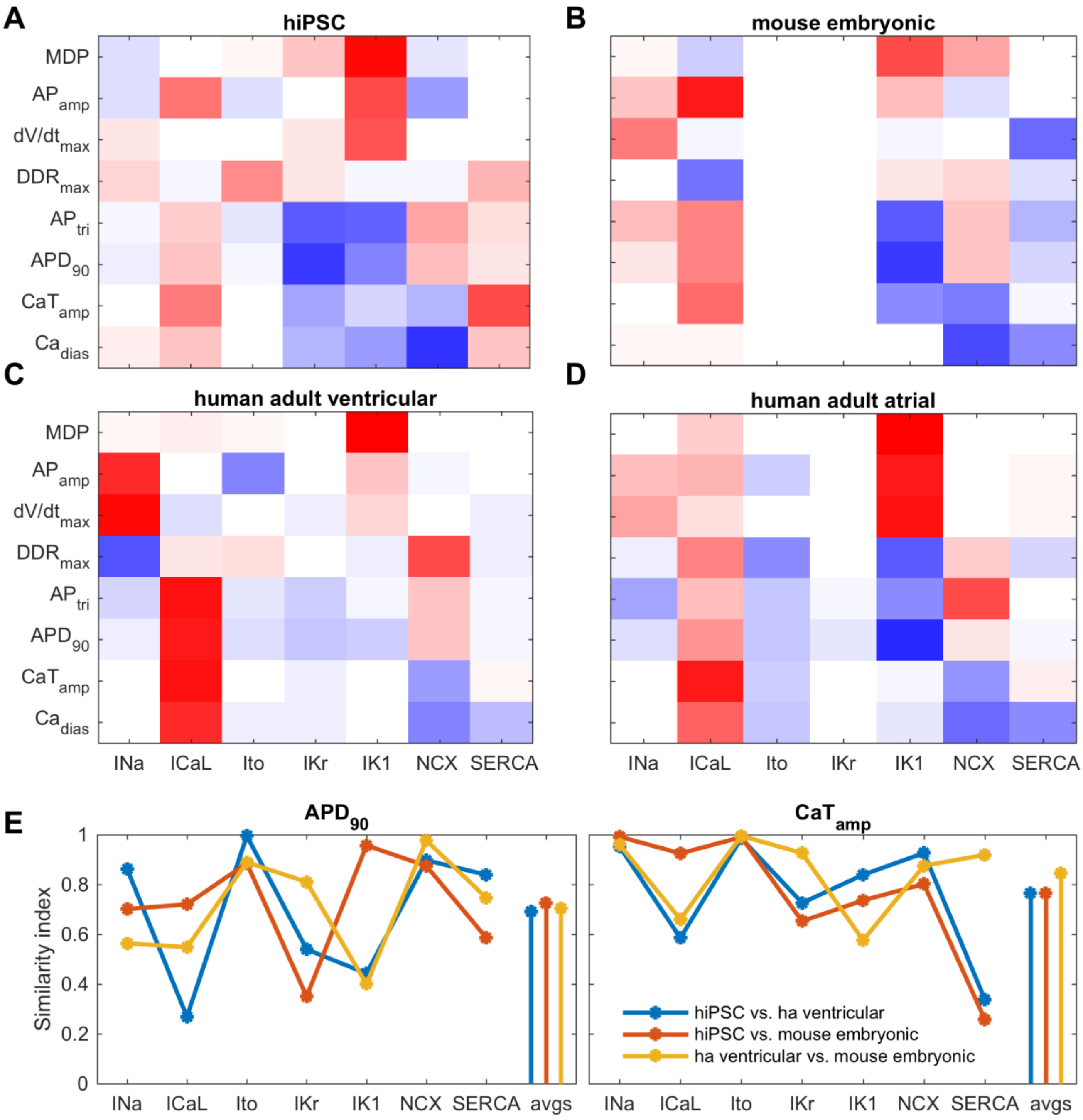

Supplementary Figure 4 | hiPSC-CM vs. human adult CM vs. mouse embryonic CM phenotype *in silico*. Correlation coefficients of sensitivity analysis for hiPSC-CM (**A**), mouse embryonic CM (**B**), human adult ventricular CM (**C**), human adult atrial CM (**D**) for APD and CaT properties vs. seven key parameters. Scale bar same as in Figure 2G. (**E**) Similarity index (absolute difference of correlation coefficients) for APD_{90} and CaT_{amp} vs. seven key biomarkers. The mouse embryonic CM model does not have I_{to} or I_{Kr}, therefore the correlation values are zeros (**B**). Simulations were run at 1 Hz pacing frequency.

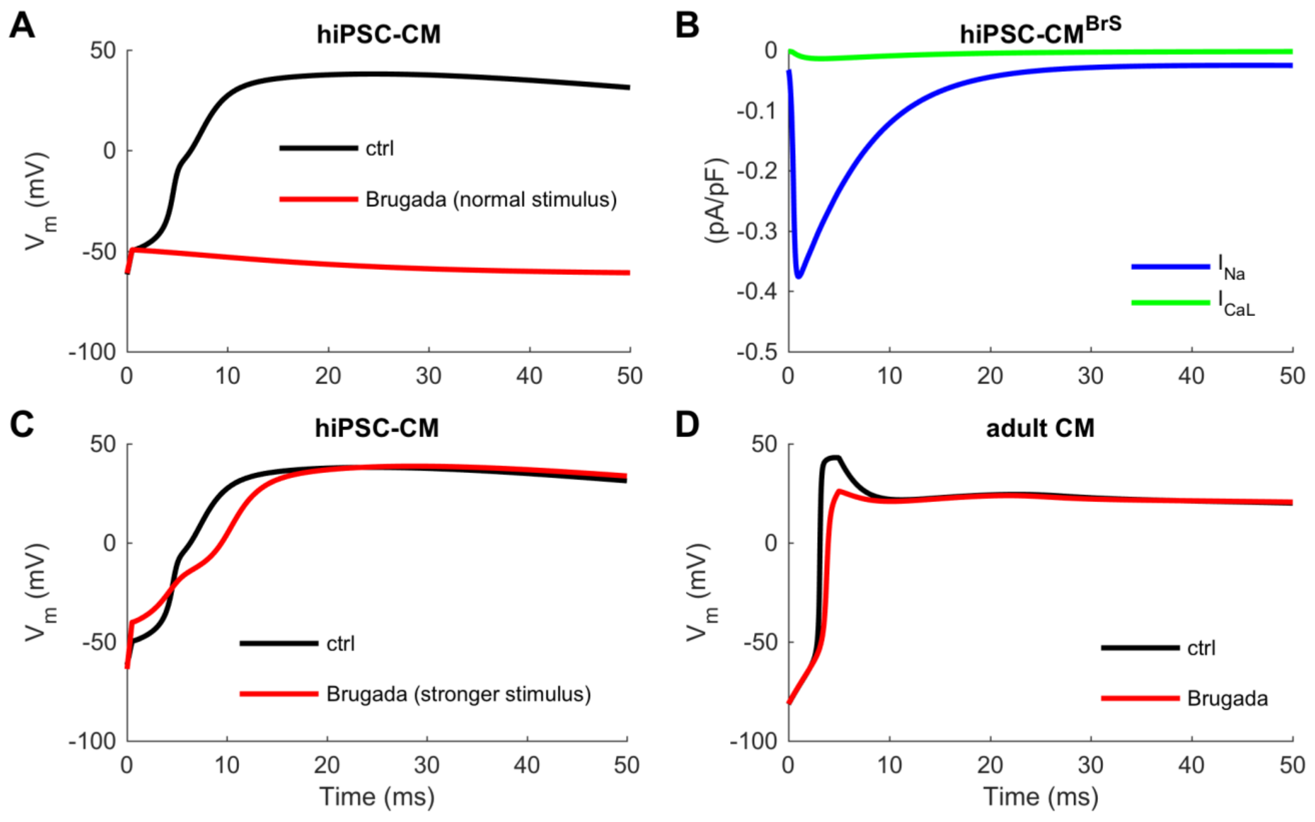

Supplementary Figure 5 | Brugada Syndrome phenotype in hiPSC-CM and adult CM *in silico*. (**A**) Failure in triggering AP in the BrS model variant. (**B**) I_{Na} and I_{CaL} are drastically smaller in BrS, as excitation threshold is increased. (**C**) Stonger stimulus current (double amplitude) elicits an AP in the BrS model variant. (**D**) In adult CM, BrS only reduces the initial AP spike and slows down slightly the AP upstroke.

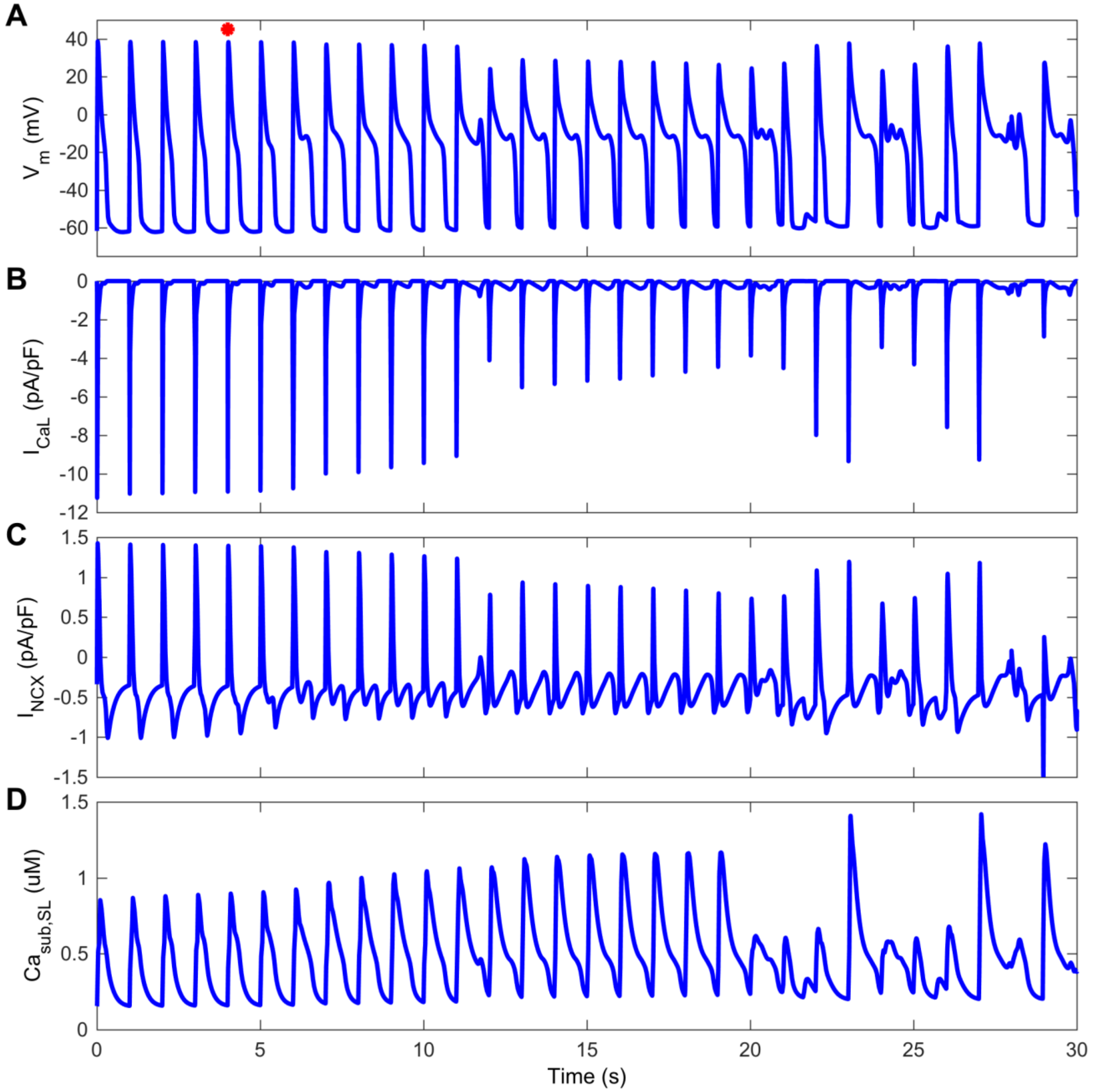

Supplementary Figure 6 | The LQT2 mutation c.A2987T KCNH2 increases arrhythmogenicity of hiPSC-CM *in silico*. Reduced repolarisation reserve (decreased I_{Kr} conductance, by 33%) increases AP duration (**A**) so much that L-type calcium channels (**B**) have enough time to recover partially and reactivate, which leads to EADs (**A**) and abnormal calcium cycling (**D**). The fifth AP, indicated by the red asterisk, was analysed in Figure 4C-D.

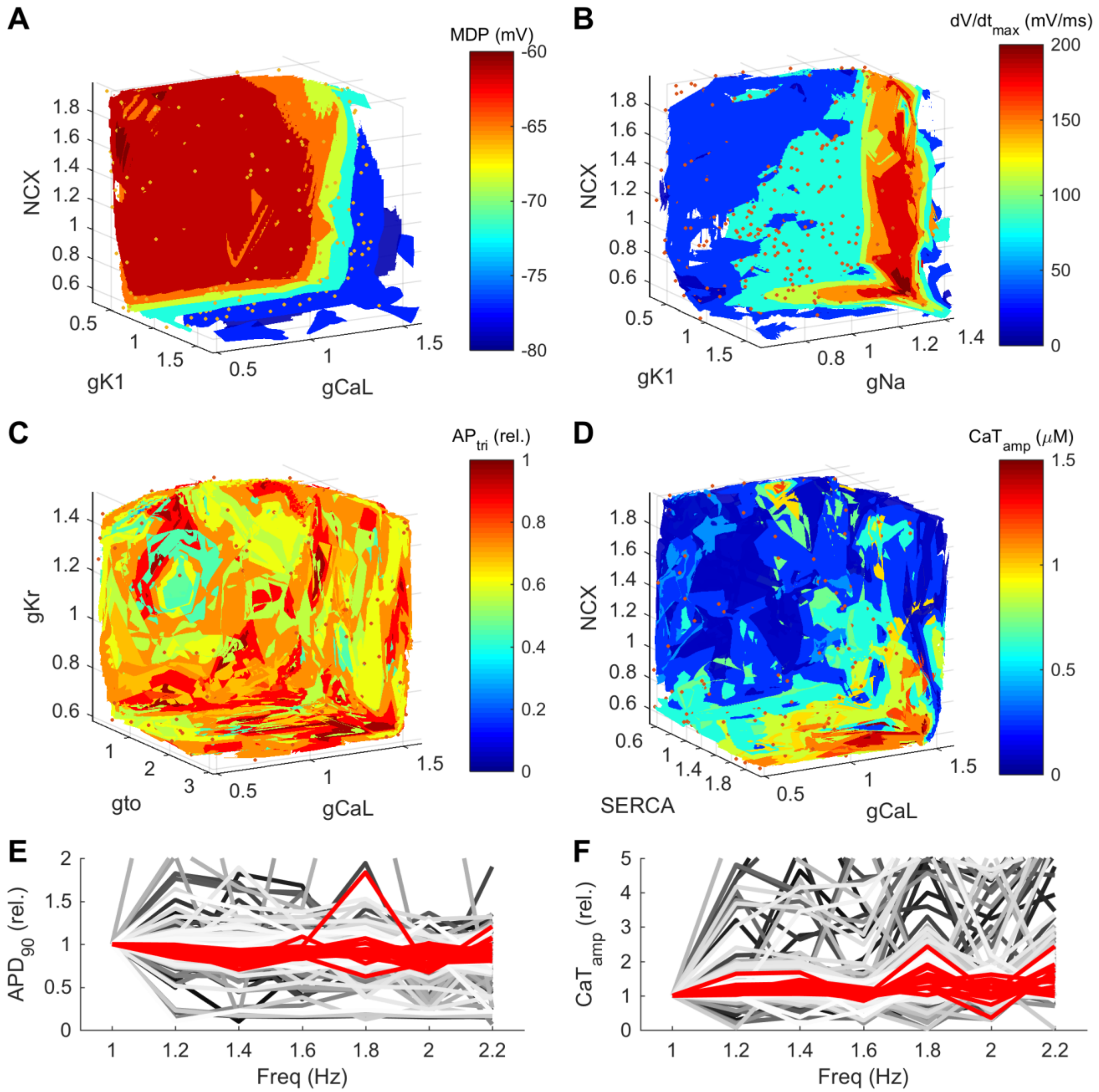

Supplementary Figure 7 | Basic properties of *in silico* hiPSC-CMs in the database. 3D surface plots of maximum diastolic potential (**A**) maximum upstroke velocity of the action potential (**B**), action potential triangulation (**C**), and calcium transient amplitude (**D**) in the *in silico* database. Restitution of APD_{90} (**E**) and force-frequency relation (**F**) in the hiPSC-CM database, results from virtual cells recapitulating both APDR and FFR highlighted with red colour.

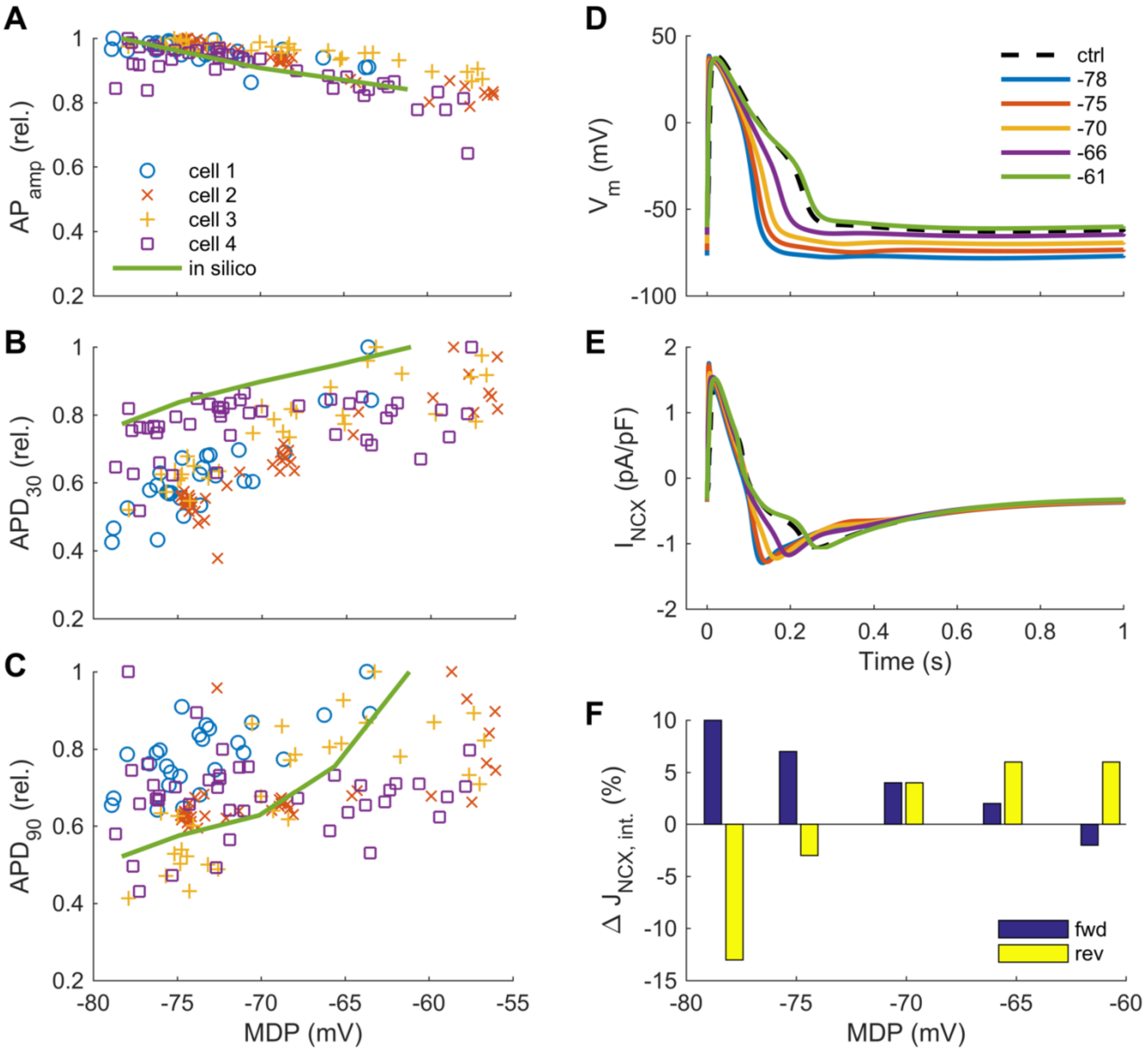

Supplementary Figure 8 | Effect of diastolic membrane potential on AP characteristics and NCX function in hiPSC-CMs. Action potential amplitude (A), duration at 30% (B) and 90% (C) repolarisation as function diastolic membrane potential *in vitro* (n=4) and *in silico*. AP morphology (D) and NCX current trace (E) for control (black dashed line) and five different membrane potentials. (F) Percent change in the integral of Ca^{2+} flux via NCX in forward (fwd) and reverse (rev) modes at five different membrane potentials, compared to control case.

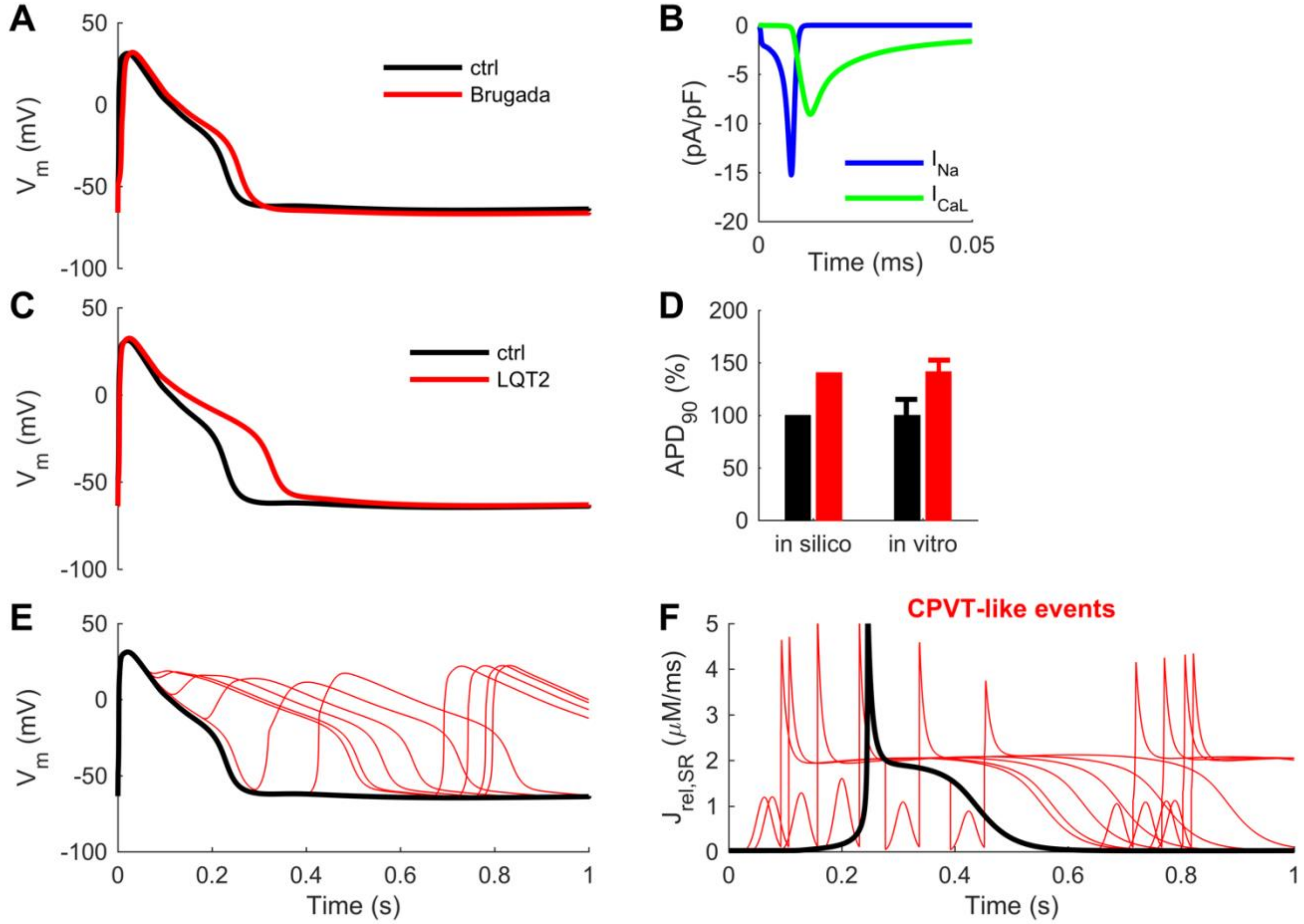

Supplementary Figure 9 | Translation of disease mechanisms in hiPSC-CM *in silico* with ideal model parameters. (**A**) Effect of Brugada-associated Na$_V$β1b/H162P mutation on AP morphology. (**B**) Sodium (blue line) and L-type calcium (green line) currents underlying the depolarisation phase of AP in BrS. Corresponding wildtype currents in hiPSC-CM are shown in Figure 2. (**C**) Effect of LQT2-associated c.A2987T KCNH2 mutation on AP repolarisation. (**D**) AP duration *in silico* and *in vitro*, as reported by Bellin et al. **(E)** Early and delayed afterdepolarisations due to CPVT-like events. **(F)** Sarcoplasmic reticulum calcium release (J_{rel}) caused by random RyR openings (CPVT-like events).

2 Supplementary Tables

Supplementary Table 1 | Ion current properties in hiPSC-CMs. LJP = liquid junction potential, nc = not corrected, nk = not known, corr = corrected.

	**hiPSC-CM type**	**Peak current (pA/pF)**	**V½ act (mV)**	**k act**	**V½ inact (mV)**	**k inact**	**Exp. conditions**	**Reference**
**I_{Na}**	*WiCell®*	−216.7 ± 18.7	−34.1	5.9	−72.1	−5.7	35–37°C, LJP:nk	(Ma et al., 2011)
	in-house	~−280 (Figure 8C)	~ −42 (Figure 8D)				37°C, LJP:nc	(Davis et al., 2012)
	WiCell®	−163.2 ± 23.8					37°C, LJP:nk	(Lee et al., 2016)
	in-house	~−118 (Figure 5B)	−42.44 ± 3.54	1.80 ± 0.67	−61.38 ± 2.51	7.58 ± 1.16	37°C, LJP:nk	(Ma et al., 2013b)
**I_{CaL}**	*WiCell*	−17.1 ± 1.7	−14.9	6.6	−29.1	−4.9	35–37°C, LJP:nk	(Ma et al., 2011)
	WiCell	−9.09 ± 0.2					34.5°C, LJP: nk	(Gibson et al., 2014)
	WiCell	−6.6 ± 2.2					37°C, LJP:nk	(Lee et al., 2016)
	in-house	−21.9 ± 1.9	-5.57	5.01	-21.28	-5.33	37°C, LJP: nc	own data (n=34)
**I_{to}**	in-house	~30 (Figure S10A)					35°C, LJP:nk	(Moretti et al., 2010)
	in-house	~0.55 (Figure 3D)					32°C, LJP:nk	(Itzhaki et al., 2011)
	WiCell	~2.4 (Figure 6D)					35–37°C, LJP:nk	(Ma et al., 2011)
	in-house	~1.9 (Figure 5C)					37°C, LJP:nk	(Lahti et al., 2012)
	WiCell	13.7 ± 1.91			−41.1 ± 0.2	6.68 ± 0.19	36°C, LJP: nk	(Cordeiro et al., 2013)
**I_{Kr}**	in-house	~1.9 (Figure 4A)					35°C, LJP:nk	(Moretti et al., 2010)
	WiCell	0.95 ± 0.02	−22.7	4.9			35–37°C, LJP:nk	(Ma et al., 2011)
	in-house	~2.5 (Figure 4D)					37°C, LJP: corr	(Bellin et al., 2013)
	WiCell	1.06 ± 0.24					36°C, LJP: nk	(Doss et al., 2012)
	in-house	1.79 ± 0.2	-26.49 ± 1.7	7.75 ± 0.7			35–37°C, LJP:nk	(Jouni et al., 2015)
	WiCell	2.3 ± 1.1					37°C, LJP:nk	(Lee et al., 2016)
	in-house	1.05 ± 0.11					36°C, LJP:corr	(Zhang et al., 2014)
**I_{Ks}**	in-house	~2.5 (Figure 4A)					35°C, LJP:nk	(Moretti et al., 2010)
	WiCell	0.31 ± 0.09					35–37°C, LJP:nk	(Ma et al., 2011)
	in-house	~0.8 (Figure 4B)			−6.5 ± 2.7	9.6 ± 2.5	37°C, LJP:nk	(Ma et al., 2015)
	WiCell	~0.6 (Figure					37°C, LJP:nk	(Ma et al., 2015)

		4B)						
	WiCell	2.9 ± 1.1					37°C, LJP:nk	(Lee et al., 2016)
	in-house	0.53 ± 0.07					36°C, LJP:corr	(Zhang et al., 2014)
**I_{K1}**	*WiCell*	−0.8 ± 0.2					35–37°C, LJP:nk	(Ma et al., 2011)
	WiCell	−2.17 ± 0.42					36°C, LJP: nk	(Doss et al., 2012)
	WiCell	−5.1 ± 1.4					37°C, LJP:nk	(Lee et al., 2016)
	in-house	−0.36 ± 0.14					35–37°C, LJP:corr	(Meijer van Putten et al., 2015)
**I_f**	*WiCell*	−4.1 ± 0.3	−84.6	8.8			35–37°C, LJP:nk	(Ma et al., 2011)
	WiCell	−0.9 ± 0.2					37°C, LJP:nk	(Lee et al., 2016)
	WiCell	~ −4.9 (Figure 7D)	−90	10.2			37°C, LJP:nk	(Wei et al., 2012)

Supplementary Table 2 | Ion transporters.

	**hiPSC-CM type**	**pump current**	**Exp. conditions**	**Reference**
**NCX**	*WiCell*®, large	4.5 ± 0.5 pA/pF		(Fine et al., 2013)
	WiCell®, small	1.8 ± 0.5 pA/pF		(Fine et al., 2013)
	in-house	2.13 ± 0.27 pA/pF	caffeine-triggered SR Ca^{2+} release, T = 22–24°C	(Zhang et al., 2013)
**NKA**	*WiCell*®, large	0.98 ± 0.1 pA/pF		(Fine et al., 2013)
	WiCell®, small	0.7 ± 2 pA/pF		(Fine et al., 2013)

Supplementary Table 3 | Cell size.

	**hiPSC-CM type**		**Reference**
**membrane area**	*WiCell*®	88.7 ± 5.0 pF	(Ma et al., 2011)
	in-house	36.0 ± 3.3 pF	(Davis et al., 2012)
	in-house	~33 ± 3 pF (Figure S11)	(Bellin et al., 2013)
	in-house	41.97 ± 4.24 pF	(Ma et al., 2013b)
	in-house	32.7 ± 2.3 pF	(Zhang et al., 2013)
	Cor.4U®	35.5 ± 27.8 pF	(Scheel et al., 2014)
	in-house	33 ± 7 pF	(Meijer van Putten et al., 2015)
	in-house	30.6 ± 2.5 pF	own data (n=88)
**width**	in-house	12.3 ± 0.5 µm	(Ma et al., 2013a)
	in-house	6.89 ± 1.79	(Gherghiceanu et al., 2011)
**length**	in-house	11.93 ± 3.99	(Gherghiceanu et al., 2011)

Supplementary Table 4 | Action potential characteristics of hiPSC-CMs *in vitro*.

hiPSC-CM type	MDP (mV)	BPM	AP_{amp} (mV)	dV/dt_{max} (mV/ms)	APD_{90} (ms)	Maturation/culturing protocol	Refs
in-house, 37°C	-76 ± 1.6	n/a	117 ± 2.8	71 ± 12	208 ± 29	20–60 days	(Bellin et al., 2013)
WiCell, 36°C	-67.0 ± 0.8	60.4 ± 2.6	101.8 ± 0.9	29.3 ± 1.7	277.3 ± 9.0	BD Matrigel™, 11-119 days	(Cordeiro et al., 2013)
in-house, 36°C	-72.4 ± 0.9	n/a	106.0 ± 3.2	115.7 ± 18.4	173.5 ± 12.2	coculture on (END-2) cells, 17 or 18 days	(Davis et al., 2012)
WiCell, 36°C	-65.9 ± 9.4	54.4 ± 30.0	103.3 ± 12.7	24.0 ± 13.8	324.8 ± 123.7	BD Matrigel™, 11–121 days	(Doss et al., 2012)
in-house, 37°C	-63.4 ± 1.3	72 ± 6	113.2 ± 2.4	26.8 ± 6.3	314.4 ± 17.6	5 weeks	(Lahti et al., 2012)
WiCell, 37°C	-66.0 ± 0.8	n/a	107.9 ± 0.8	44.5 ± 2.8	492.1 ± 15.4	4 weeks	(Lee et al., 2016)
in-house, 36°C	-75.6 ± 1.2	35 ± 2	104.0 ± 1.1	27.8 ± 4.8	414.7 ± 21.8	30–32 days	(Ma et al., 2011)
in-house, 37°C	-61.4 ± 1.4	69.1 ± 11.3	86.0 ± 1.4	13.1 ± 4.7	434.0 ± 31.1	30–32 days	(Ma et al., 2013b)
WiCell®, 37°C	-59.8 ± 0.7	71.0 ± 5.2	99.1 ± 2.6	n/a	372.9 ± 14.2	4–5 weeks	(Ma et al., 2015)
in-house, 35-37°C	-56 ± 4	n/a	73 ± 9	10 ± 7	162 ± 27	30 days	(Meijer van Putten et al., 2015)
in-house, 37°C	-58 ± 4	38.8 ± 3.6	100 ± 2	6.3	n/a	25–43 days	(Novak et al., 2012)
in silico		45.1					own data

Supplementary Table 5 | Calcium transient characteristics. Measured at either 0.5 or 1 Hz pacing.

hiPSC-CM type	Time to peak (ms)	Time to 50%, 66% and 90% decay (ms)			Decay time constant (ms)	Maturation protocol, time (d)	Ref
WiCell®, Wisconsin	144	224	n/a	505	n/a	microgrooved PDMS culture, -	(Rao et al., 2013)
WiCell®, Wisconsin	200	258	n/a	495	n/a	unstructured, -	(Rao et al., 2013)
SCVI, Stanford	513	n/a	n/a	n/a	967	Small Molecule Induction, 30	(Hwang et al., 2015)
Vanderbilt	562	n/a	n/a	n/a	1166	Matrigel Sandwich Induction, 30	(Hwang et al., 2015)
WiCell®, Wisconsin	586	n/a	n/a	n/a	985	Matrigel Sandwich Induction, 30	(Hwang et al., 2015)
WiCell®, Wisconsin	n/a	168	n/a	n/a	n/a	Matrigel (BD Biosciences), -	(Chen et al., 2015)

in-house	86	n/a	181.8 ± 6.2	n/a	n/a	Please, see Materials and Methods section.	own data (n=36)

3 Supplementary References

Bellin, M., Casini, S., Davis, R. P., D'Aniello, C., Haas, J., Ward-van Oostwaard, D., et al. (2013). Isogenic human pluripotent stem cell pairs reveal the role of a KCNH2 mutation in long-QT syndrome: Isogenic pairs of LQT2 pluripotent stem cells. *EMBO J.* 32, 3161–3175. doi:10.1038/emboj.2013.240.

Chen, G., Li, S., Karakikes, I., Ren, L., Chow, M. Z.-Y., Chopra, A., et al. (2015). Phospholamban as a Crucial Determinant of the Inotropic Response of Human Pluripotent Stem Cell–Derived Ventricular Cardiomyocytes and Engineered 3-Dimensional Tissue Constructs. *Circ. Arrhythm. Electrophysiol.* 8, 193–202. doi:10.1161/CIRCEP.114.002049.

Cordeiro, J. M., Nesterenko, V. V., Sicouri, S., Goodrow, R. J., Treat, J. A., Desai, M., et al. (2013). Identification and characterization of a transient outward K+ current in human induced pluripotent stem cell-derived cardiomyocytes. *J. Mol. Cell. Cardiol.* 60, 36–46. doi:10.1016/j.yjmcc.2013.03.014.

Davis, R. P., Casini, S., Berg, C. W. van den, Hoekstra, M., Remme, C. A., Dambrot, C., et al. (2012). Cardiomyocytes Derived From Pluripotent Stem Cells Recapitulate Electrophysiological Characteristics of an Overlap Syndrome of Cardiac Sodium Channel Disease. *Circulation* 125, 3079–3091. doi:10.1161/CIRCULATIONAHA.111.066092.

Doss, M. X., Di Diego, J. M., Goodrow, R. J., Wu, Y., Cordeiro, J. M., Nesterenko, V. V., et al. (2012). Maximum Diastolic Potential of Human Induced Pluripotent Stem Cell-Derived Cardiomyocytes Depends Critically on IKr. *PLoS ONE* 7, e40288. doi:10.1371/journal.pone.0040288.

Fine, M., Lu, F.-M., Lin, M.-J., Moe, O., Wang, H.-R., and Hilgemann, D. W. (2013). Human-induced pluripotent stem cell-derived cardiomyocytes for studies of cardiac ion transporters. *Am. J. Physiol. - Cell Physiol.* 305, C481–C491. doi:10.1152/ajpcell.00143.2013.

Gherghiceanu, M., Barad, L., Novak, A., Reiter, I., Itskovitz-Eldor, J., Binah, O., et al. (2011). Cardiomyocytes derived from human embryonic and induced pluripotent stem cells: comparative ultrastructure. *J. Cell. Mol. Med.* 15, 2539–2551. doi:10.1111/j.1582-4934.2011.01417.x.

Gibson, J. K., Yue, Y., Bronson, J., Palmer, C., and Numann, R. (2014). Human stem cell-derived cardiomyocytes detect drug-mediated changes in action potentials and ion currents. *J. Pharmacol. Toxicol. Methods* 70, 255–267. doi:10.1016/j.vascn.2014.09.005.

Hwang, H. S., Kryshtal, D. O., Feaster, T. K., Sánchez-Freire, V., Zhang, J., Kamp, T. J., et al. (2015). Comparable calcium handling of human iPSC-derived cardiomyocytes generated by multiple laboratories. *J. Mol. Cell. Cardiol.* 85, 79–88. doi:10.1016/j.yjmcc.2015.05.003.

Itzhaki, I., Maizels, L., Huber, I., Zwi-Dantsis, L., Caspi, O., Winterstern, A., et al. (2011). Modelling the long QT syndrome with induced pluripotent stem cells. *Nature* 471, 225–229. doi:10.1038/nature09747.

Jouni, M., Si-Tayeb, K., Es-Salah-Lamoureux, Z., Latypova, X., Champon, B., Caillaud, A., et al. (2015). Toward Personalized Medicine: Using Cardiomyocytes Differentiated From Urine-Derived Pluripotent Stem Cells to Recapitulate Electrophysiological Characteristics of Type 2 Long QT Syndrome. *J. Am. Heart Assoc.* 4, e002159. doi:10.1161/JAHA.115.002159.

Lahti, A. L., Kujala, V. J., Chapman, H., Koivisto, A.-P., Pekkanen-Mattila, M., Kerkelä, E., et al. (2012). Model for long QT syndrome type 2 using human iPS cells demonstrates arrhythmogenic characteristics in cell culture. *Dis. Model. Mech.* 5, 220–230. doi:10.1242/dmm.008409.

Lee, S., Lee, H.-A., Choi, S. W., Kim, S. J., and Kim, K.-S. (2016). Evaluation of nefazodone-induced cardiotoxicity in human induced pluripotent stem cell-derived cardiomyocytes. *Toxicol. Appl. Pharmacol.* 296, 42–53. doi:10.1016/j.taap.2016.01.015.

Ma, D., Wei, H., Lu, J., Ho, S., Zhang, G., Sun, X., et al. (2013a). Generation of patient-specific induced pluripotent stem cell-derived cardiomyocytes as a cellular model of arrhythmogenic right ventricular cardiomyopathy. *Eur. Heart J.* 34, 1122–1133. doi:10.1093/eurheartj/ehs226.

Ma, D., Wei, H., Lu, J., Huang, D., Liu, Z., Loh, L. J., et al. (2015). Characterization of a novel KCNQ1 mutation for type 1 long QT syndrome and assessment of the therapeutic potential of a novel IKs activator using patient-specific induced pluripotent stem cell-derived cardiomyocytes. *Stem Cell Res. Ther.* 6, 39. doi:10.1186/s13287-015-0027-z.

Ma, D., Wei, H., Zhao, Y., Lu, J., Li, G., Sahib, N. B. E., et al. (2013b). Modeling type 3 long QT syndrome with cardiomyocytes derived from patient-specific induced pluripotent stem cells. *Int. J. Cardiol.* 168, 5277–5286. doi:10.1016/j.ijcard.2013.08.015.

Ma, J., Guo, L., Fiene, S. J., Anson, B. D., Thomson, J. A., Kamp, T. J., et al. (2011). High purity human-induced pluripotent stem cell-derived cardiomyocytes: electrophysiological properties of action potentials and ionic currents. *Am. J. Physiol. - Heart Circ. Physiol.* 301, H2006–H2017. doi:10.1152/ajpheart.00694.2011.

Meijer van Putten, R. M. E., Mengarelli, I., Guan, K., Zegers, J. G., van Ginneken, A. C. G., Verkerk, A. O., et al. (2015). Ion channelopathies in human induced pluripotent stem cell derived cardiomyocytes: a dynamic clamp study with virtual IK1. *Membr. Physiol. Membr. Biophys.* 6, 7. doi:10.3389/fphys.2015.00007.

Moretti, A., Bellin, M., Welling, A., Jung, C. B., Lam, J. T., Bott-Flügel, L., et al. (2010). Patient-Specific Induced Pluripotent Stem-Cell Models for Long-QT Syndrome. *N. Engl. J. Med.* 363, 1397–1409. doi:10.1056/NEJMoa0908679.

Novak, A., Barad, L., Zeevi-Levin, N., Shick, R., Shtrichman, R., Lorber, A., et al. (2012). Cardiomyocytes generated from CPVTD307H patients are arrhythmogenic in response to β-

adrenergic stimulation. *J. Cell. Mol. Med.* 16, 468–482. doi:10.1111/j.1582-4934.2011.01476.x.

Paci, M., Sartiani, L., Lungo, M. D., Jaconi, M., Mugelli, A., Cerbai, E., et al. (2012). Mathematical modelling of the action potential of human embryonic stem cell derived cardiomyocytes. *Biomed. Eng. OnLine* 11, 61. doi:10.1186/1475-925X-11-61.

Rao, C., Prodromakis, T., Kolker, L., Chaudhry, U. A. R., Trantidou, T., Sridhar, A., et al. (2013). The effect of microgrooved culture substrates on calcium cycling of cardiac myocytes derived from human induced pluripotent stem cells. *Biomaterials* 34, 2399–2411. doi:10.1016/j.biomaterials.2012.11.055.

Sartiani, L., Bettiol, E., Stillitano, F., Mugelli, A., Cerbai, E., and Jaconi, M. E. (2007). Developmental Changes in Cardiomyocytes Differentiated from Human Embryonic Stem Cells: A Molecular and Electrophysiological Approach. *STEM CELLS* 25, 1136–1144. doi:10.1634/stemcells.2006-0466.

Scheel, O., Frech, S., Amuzescu, B., Eisfeld, J., Lin, K.-H., and Knott, T. (2014). Action Potential Characterization of Human Induced Pluripotent Stem Cell–Derived Cardiomyocytes Using Automated Patch-Clamp Technology. *ASSAY Drug Dev. Technol.* 12, 457–469. doi:10.1089/adt.2014.601.

Wei, H., Zhang, G., Qiu, S., Lu, J., Sheng, J., Manasi, et al. (2012). Hydrogen Sulfide Suppresses Outward Rectifier Potassium Currents in Human Pluripotent Stem Cell-Derived Cardiomyocytes. *PLOS ONE* 7, e50641. doi:10.1371/journal.pone.0050641.

Yaniv, Y., Maltsev, V. A., Ziman, B. D., Spurgeon, H. A., and Lakatta, E. G. (2012). The “Funny” Current (If) Inhibition by Ivabradine at Membrane Potentials Encompassing Spontaneous Depolarization in Pacemaker Cells. *Molecules* 17, 8241–8254. doi:10.3390/molecules17078241.

Zhang, M., D’Aniello, C., Verkerk, A. O., Wrobel, E., Frank, S., Oostwaard, D. W., et al. (2014). Recessive cardiac phenotypes in induced pluripotent stem cell models of Jervell and Lange-Nielsen syndrome: Disease mechanisms and pharmacological rescue. *Proc. Natl. Acad. Sci.* 111, E5383–E5392. doi:10.1073/pnas.1419553111.

Zhang, X.-H., Haviland, S., Wei, H., Šarić, T., Fatima, A., Hescheler, J., et al. (2013). Ca2+ signaling in human induced pluripotent stem cell-derived cardiomyocytes (iPS-CM) from normal and catecholaminergic polymorphic ventricular tachycardia (CPVT)-afflicted subjects. *Cell Calcium* 54, 57–70. doi:10.1016/j.ceca.2013.04.004.
